# Polyphenolic Screening and the Antioxidant Activity of Grape Pomace Extracts of Romanian White and Red Grape Varieties

**DOI:** 10.3390/antiox13091133

**Published:** 2024-09-19

**Authors:** Cristiana Radulescu, Radu Lucian Olteanu, Claudia Lavinia Buruleanu, Mihaela Nechifor (Tudorache), Ioana Daniela Dulama, Raluca Maria Stirbescu, Ioan Alin Bucurica, Sorina Geanina Stanescu, Andreea Laura Banica

**Affiliations:** 1 Faculty of Sciences and Arts, Valahia University of Targoviste, 13 Sinaia Alley, 130004 Targoviste, Romania; 2 Doctoral School Chemical Engineering and Biotechnology, National University of Science and Technology Politehnica of Bucharest, 313 Splaiul Independentei, 060042 Bucharest, Romania; tudorache.mihaela-db@ansvsa.ro (M.N.); banica.andreea@icstm.ro (A.L.B.); 3 Academy of Romanian Scientists, 3 Ilfov, 050044 Bucharest, Romania; 4 Faculty of Environmental Engineering and Food Science, Valahia University of Targoviste, 13 Sinaia Alley, 130004 Targoviste, Romania; 5 Institute of Multidisciplinary Research for Science and Technology, Valahia University of Targoviste, 13 Sinaia Alley, 130004 Targoviste, Romania; dulama.ioana@icstm.ro (I.D.D.); stirbescu.raluca@icstm.ro (R.M.S.); bucurica_alin@icstm.ro (I.A.B.); geanina.stanescu@icstm.ro (S.G.S.)

**Keywords:** grape pomace, hydroalcoholic extract, polyphenols, antioxidant activity, anthocyanins, tannins, HPLC

## Abstract

Due to its valuable organic compounds, grape pomace represents a valuable resource in the creation of value-added food products. In this study, we investigated grape pomace hydroalcoholic extracts obtained by ultrasonication from two white and two red Romanian grape varieties. The phytochemical parameters, i.e., polyphenolics, flavonoids, anthocyanins, condensed tannins content, and antioxidant activity, were determined by UV-Vis spectrometry. The statistical analysis revealed that Tămâioasă Românească and Negru de Drăgășani, respectively, and Fetească Albă and Fetească Neagră are similar to each other. Significant differences among the pomaces obtained from different grape varieties were noticed in terms of their phenolic contents. The red varieties (Fetească Neagră and Negru de Drăgășani) were richest in terms of total phenolics and total anthocyanins, respectively. The antioxidant activity of extracts obtained from grape pomace showed that Negru de Drăgășani had the higher value due to its high amounts of polyphenols, followed by Fetească Neagră, Fetească Albă, and Tămâioasă Românească. Higher levels of bioactive polyphenolic substances, i.e., catechin, myricetin, resveratrol, and vanillic acid, were found in the pomace of Negru de Drăgășani extracts using high-performance liquid chromatography. These results emphasize the potential of the Negru de Drăgășani variety to be further exploited for nutritional and functional applications.

## 1. Introduction

The wine industry generates significant amounts of by-products, which can lead to the worsening of ecological problems. Over 75 million tons of grapes are harvested worldwide, of which 75% are used in winemaking, producing approximately 38 billion liters of wine annually [1]. The remaining skin, seeds, and stalks, namely, 20–25% of the total grape weight used for winemaking, represent secondary products, which are still very poorly exploited in the context of the circular economy [2,3,4]. Currently, grape pomace is used for animal feed, waste-based compost, and to improve thermal isolation in building construction [4].

Meanwhile, on a global level, solutions are being sought for the valorization of wine by-products, such as the recovery, bioconversion, and use of their valuable biocompounds. This results in products with added value being obtained, such as extracts of bioactive compounds, grape seed flour, grape skin flour, grape seed extracts, grape seed oil, wine colorants, and so on [5,6].

Grape pomace contains a variety of polyphenols (i.e., flavonoids, such as anthocyanidins, flavonol, flavanol, and tannins, and non-flavonoids, such as phenolic acids derived from cinnamic and benzoic acids and stilbenes), which are extracted using environmentally friendly solvents such as ethanol, glycerol, and water. These extracts are used in the food and cosmetic industries, being added to various products to improve antioxidant activity and protect the skin from the harmful effects of ultraviolet radiation. In addition, Cotoras et al. mentioned that red grape pomace contains high amounts of resveratrol and other polyphenols such as the flavanols myricetin, quercetin, and kaempferol; the flavan-3-ols catechin and epicatechin; the cinnamic acid *p*-coumaric; benzoic acids, such as syringic, gallic, and protocatechuic; and 4-hydroxybenzoic acids [7].

The polyphenols present in grape pomace have demonstrated anticancer properties [8,9,10,11,12,13] and provide cardioprotective benefits by improving vasorelaxation and lipid profiles [8,9]. In the context of diabetes, certain compounds found in grape pomace inhibit the digestive enzymes involved in the breakdown of carbohydrates [14,15,16,17]. On the other hand, these compounds can improve the plasma lipid profile and reduce blood triglycerides [18,19,20].

In addition, the supplementation of foods with pomace promotes antioxidant activity and improves the nutritional profile of baked goods, black tea, and chocolate [21,22]. Adding microencapsulated powders containing grape pomace extracts to food can extend the shelf life and inhibit the activity of harmful bacteria while promoting the probiotic activity of beneficial bacteria [23,24]. The addition of nano-emulsion-based capsules, produced from grape pomace phenolic compounds, to hazelnut paste inhibits unwanted oxidation [5,25]. Recently, a study was carried out to experimentally produce vegetarian sausages without the application of heat treatment and with the use of grape flour [26].

The grape by-products used to enhance foods contribute to the nutritional and functional value of products by increasing the fiber content and the presence of antioxidant compounds [27]. The remaining question is whether this improvement has health benefits [23,28]. The health benefits of grape by-products can also be determined by observing the phenolic compounds [24,29,30] that can potentially be recovered from them. The amount and type of phenolic compounds present in grape pomace depend on the variety, the climatic conditions, and the applied extraction technique.

In the current study, the phytochemical screening (total polyphenols content, total flavonoids content, total content of anthocyanins, and condensed tannins content) of grape pomace hydroalcoholic extracts of four Romanian grape varieties (i.e., white Fetească Albă and Tămâioasă Românească, and red Fetească Neagră and Negru de Drăgășani) was investigated for the first time. Statistical analysis identified strong correlations between the antioxidant activity, phytochemicals content, and polyphenols (i.e., catechin, vanillic acid, caffeic acid, myricetin, resveratrol, and kaempferol) of grape pomaces. Avoiding a high number of experiments due to their natural variability, these results can be further used if the valorization of grape pomaces is pursued for different applications generated by a multitude of influencing factors.

## 2. Materials and Methods

### 2.1. Site Description

**Figure 1:**
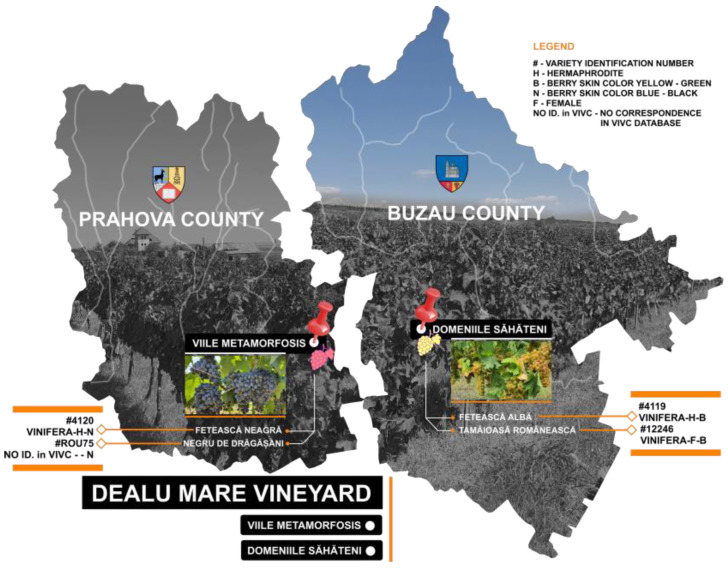
Dealu Mare vineyard, according to REVINO Gourmet 2023 [33].

Native grape samples (i.e., two white varieties, namely, Fetească Albă and Tămâioasă Românească, and two red varieties, namely, Fetească Neagră and Negru de Drăgășani) were collected from one of the most known vineyards in Romania, situated in the middle of the Dealu Mare Region (Figure 1). This site is known for the quality of its grapes and wine production in terms of European norms regarding organic cultures. The vineyard is located on the hills around the Carpathian Mountains. These places benefit from different types of calcareous soil/subsoil and a continental climate, with thermal amplitudes and long and sunny summers favoring good grape maturity [22,31,32].

### 2.2. Materials and Reagents

Grape samples of four native grape varieties (i.e., two white varieties Fetească Albă and Tămâioasă Românească, and two red varieties Fetească Neagră and Negru de Drăgășani) were collected in September 2023, according to their maturity parameters (i.e., sugar content, titratable acidity, and pH) [34] determined by the specialists from Domeniile Săhăteni (for white grapes) and Viile Metamorphosis (for red grapes). Representative portions of each sample were used for further analyses [34]. A brief description of the botanical characteristics and phytochemical profiles of the native white and red grape varieties is provided as follows.

Fetească Albă variety #4119 vinifera-H-B (# variety identification number, species, sex (H = hermaphrodite), berry skin color B = yellow-green [35]) is thousands of years old [36]. *Botanical characteristics*: compact grape with medium-to small-sized berries; grape average weight is 90–200 g; 10–15 cm long; short and lignified stem; vigorous and resistant to cold weather; round yellow-green berries with visible pistillate points; number of grains/bunches ranged from 45 to 122; skin-thin yellow-green with a visible pistillate point, covered with plum, rich in tannins and sugars; three seeds, hard outer shell, brown, approx. 2–3% of the weight of berries. *Phytochemical profile*: grape seed contains 7–20% oil (dry basis); pulp is juicy with a citrus taste and contains 180–200 g/L of sugars. Grape pomace is yellow, with 3.8–4.2 g/L acidity and 200–230 g/L of sugars and citrus flavor [22,31].

Tămâioasă Românească variety #12246 vinifera-F-B (# variety identification number, species, sex (H = hermaphrodite), berry skin color B = yellow-green [35]) has been cultivated for more than two millennia in the Romanian vineyards [22,31]. *Botanical characteristics*: branched bunches, lengths ranging between 15 and 25 cm, medium weight of 300 g; dense, spherical berries, crunchy, juicy core, and an intense incense taste; 0.90–1.20 cm diameter; green-yellow to amber appearance when ripe; the number of grains per bunch ranged from 81 to 145; thin skin covered with plum in a thick layer; two or three seeds, hard outer shell, brown. *Phytochemical profile*: 240–250 g/L to 270–300 g/L sugars in favorable years; skin rich in tannins and sugar; pulp is juicy, with an intense incense taste. Grape pomace is a yellow-amber color, with an acidity of 3.4–4.0 g/L and containing 240–260 g/L of sugars and incense flavor [22,31].

Fetească Neagră variety #4120 vinifera-H-N/ROU-2009 -W (# variety identification number, species, sex (H = hermaphrodite), berry skin color N = blue-black [35]) is very old, known since the time of the Dacians [37,38,39]. *Botanical characteristics*: grape bunches are 14 cm long, cylindrical in shape, sometimes conical-cylindrical, with dense berries; the diameter of ripe grape berries is 1.2–1.5 cm; the vine is vigorous, reddish-brown at the nodes, and coppery to yellowish in the internodes. The grape pomace is red, with 4.0–4.8 g/L acidity and 200–220 g/L of sugars. *Phytochemical characteristics*: a high concentration of sugars of approximately 240 g/L; strong aroma of ripe plum, honey, and spices; content of fine tannins [24]; antioxidant activity (AA) 14.81 ± 3.96 mg equivalent ascorbic acid/g (Prieto method), total polyphenols content (TPC) 106.46 ± 6.21 mg gallic acid/mL, and total flavonoid content (TFC) 99.50 ± 4.22 mg equivalent quercetin/g, values highlighted in several research and patents by Radulescu et al. [11,38,39].

Negru de Drăgășani variety # ROU75 no correspondence in VIVC database—N (# variety identification number, species, berry skin color N = blue-black [35]). *Botanical characteristics*: grape is medium in size, cylindrical-conical, uniaxial, bi- or tri-winged; berries are medium (2.0–2.5 g) ovoid in shape, have a bluish-black color, and are covered with pruina; the pulp is red, juicy, unflavored and with a frank taste. Phytochemical characteristics: grape red-black color, 233–234 g/L sugar, and 4.5 g/L acidity (H_2_SO_4_), with a high anthocyanin and tannin content.

All reagents (Merck KGaA and Carl Roth, Germany, and Sigma-Aldrich, Saint Louis, USA) were of HPLC grade, and the liquid samples were filtered before use to avoid the accidental contamination of samples (including ultrapure water) or the risk of possible interference in physicochemical analyses. Deionized water (with conductivity below 0.5 µS/cm at 25 °C) was used as a solvent and as a wash solution.

### 2.3. Sample Preparation

Grape pomace was obtained in the laboratory, following the stages of the initial fermentation process, for both white and red varieties. The collected grapes were checked from an ampelographic point of view (each bunch was measured, weighed, and the berries’ size was measured as well), then they were washed, separated from the bunches grain by grain, pressed, then placed and kept in a fermentation vessel for seven days. Several parameters such as pH, acidity, and refractive index were determined during this time. Finally, the grape pomace (skin and seeds) was lyophilized and stored in the refrigerator (4 °C) for the subsequent analyses.

#### 2.3.1. Preparation of Grape Pomace Extract by Ultrasonication

The lyophilized plant material (pomace) was weighed (~1.00 g) into 15 mL brown-walled capped vials using an analytical balance, over which was added 10 mL of extraction solvent, i.e., absolute ethyl alcohol (99–100%):water = 1:1; thus, duplicate extracts of each variety were obtained. The extraction itself was carried out in two stages: (1) ultrasonication for 20 min (initial temperature 29 °C and final temperature 39 °C) with intermittent agitation (vortex) every 5 min; the supernatant was separated by centrifugation at room temperature (10 min/5000 rpm); (2) 10 mL of extraction solvent was added to the precipitate from the vials and was followed by ultrasonication for 20 min (initial temperature 35 °C and final temperature 45 °C) with intermittent agitation (vortex) every 5 min; the supernatant was separated by centrifugation at room temperature (10 min/6000 rpm) and mixed with the supernatant collected from extraction step 1. The supernatant resulted from the two extraction steps was filtered (PRAT DUMAS France/Whatman equivalence 42, micrometric retention 7–9 mm) and placed into a 25 mL volumetric flask (with brown walls) using an extraction solvent. The extracts obtained by this method [40,41] were stored in the refrigerator (4 °C) until further analyses.

#### 2.3.2. Samples Preparation for High-Performance Liquid Chromatography

The extracts obtained according to subsection 2.3.1 were analyzed using high-performance liquid chromatography (HPLC) with fluorescence detection (FLD) and diode array detection (DAD). The HPLC method was developed and optimized for the detection and separation of six polyphenols (i.e., catechin, vanillic acid, caffeic acid, myricetin, resveratrol, and kaempferol). The stock solution containing the six polyphenols with a concentration of 100 ppm was prepared by adding aliquots (50–200 μL, Table S1) from individual stocks, and was then brought to 1000 μL using methanol. The calibration solutions in the range of 0.1–5.0 ppm were prepared by successive dilutions from the stock solution.

### 2.4. Phytochemical Analyses

#### 2.4.1. DPPH Radical Scavenging Assay

The method is based on the discoloration/decrease in the absorbance of the free radical DPPH (2,2-diphenyl-picryl-hydrazyl), which is an intense red-purple/violet color (having an intense absorption band at 517 nm), by substances with antioxidant activity. Preparation of the DPPH solution [42]: 7.89 mg of DPPH was weighed and dissolved in a 100 mL volumetric flask using ethanol HPLC grade (0.2 mM DPPH); the solution was kept in the dark for 2 h until the absorbance had stabilized. After 2 h of preparation, 1 mL of DPPH solution was taken in a test tube/vial and 0.2 mL of ethanol and 0.8 mL of 2-amino-2-(hydroxymethyl) propane-1,3-diol (Tris)-HCl buffer solution (pH=7.4) were added. After homogenization (vortex), the absorbance (λ = 517 nm) was measured using a blank (i.e., a mixture of 1.2 mL ethanol and 0.8 mL Tris-HCl buffer solution, pH=7.4). If the absorbance was in the range of 1.00 ± 0.05, the DPPH solution was used directly for analysis; if the absorbance exceeded the value of 1.05, it was diluted with ethanol to correct it to 1.00 ± 0.05. DPPH assay procedure. In a test tube, a 0.2 mL extract sample was taken, then 0.8 mL Tris-HCl buffer solution and 1 mL DPPH solution were added and then homogenized (vortex) for 10 s. The prepared samples were kept in the dark at room temperature for 30 min, then the absorbance of the sample and control sample (A_sample_ and A_control_) were measured at 517 nm using an Evolution 260 Bio UV-Visible spectrophotometer (Thermo Fisher Scientific Inc., Madison, WI, USA). The control sample was a mixture of 0.2 mL ethanol, 0.8 mL Tris-HCl buffer solution, and 1 mL DPPH solution.

The Inhibition Ratio, IR (%) was calculated with Equation (1) [42]:

(1) $$IR\left(\%\right)=\left[\left(A_{control}\right.-\left.A_{sample}\right)/A_{control}\right]\cdot 100$$

All samples were analyzed in triplicate. Gallic acid was used for the calibration curve in order to obtain the reference value necessary to decrease the initial DPPH• absorbance by 50% (IC_50_, μg GAE/mL); the amount of antioxidants was expressed as gallic acid equivalents (GAE) [42,43].

#### 2.4.2. Total Polyphenols Content

The method is based on the oxidation of the substances using Folin–Ciocalteu reagent in basic medium [44]. The applied procedure involved several steps: (1) 1 mL of pomace extract was diluted with distilled water in a 10 mL volumetric flask; (2) 0.5 mL of the diluted extract was taken, to which 2.5 mL of Folin–Ciocalteu reagent 10% was added; (3) the mixture was stirred for 8 min; (4) 2 mL Na_2_CO_3_ 8% was added and stirred (vortex) for 1 min. The blank was prepared similarly with 0.5 mL distilled water. The extract samples and the blank were maintained for 60 min at room temperature. Absorbance (A) of the samples against the blank was measured at 765 nm, using an Evolution 260 Bio UV-Visible spectrophotometer (Thermo Fisher Scientific Inc., Madison, WI, USA). The total polyphenols content (TPC) was determined based on the calibration curve (gallic acid—GA, Carl Roth GmbH and Co. KG, Karlsruhe-Mühlburg, Germany) considering the dilution ratio of the extract. The results are expressed in mg GAE/mL (mg gallic acid equivalents/mL extract) and mg GAE/g dry plant material (DW), respectively.

#### 2.4.3. Total Flavonoids Content

The method is based on the reaction between flavonoids and aluminum chloride (AlCl_3_·6H_2_O) in a weak basic medium with the formation of a yellow-orange colored complex quantified colorimetrically at 510 nm. The procedure was achieved in the presence of sodium nitrite in a basic medium, which suggests that it is specific to rutin, luteolin, and catechins (phenolic acids also show a notable absorbance at 510 nm) [45]. The aluminum chloride assay involved the following steps: (1) a 0.3 mL solution of 5% NaNO_2_ was added to 1 mL of extract sample and 4 mL of distilled water; (2) the mixture was stirred and allowed to rest for 5 min; (3) 0.3 mL of 10% AlCl_3_ solution was added; (4) the mixture was stirred (vortex) and maintained at rest for 5 min; (5) 2 mL of 1 M NaOH solution was added and stirred again. The mixture was brought to a final volume of 10 mL by adding 2.4 mL of distilled water and stirred again. The blank was prepared using 1 mL of distilled water instead of extract. Analysis was achieved in triplicate. Absorbance (A) of the samples and the blank was measured at 510 nm, using an Evolution 260 Bio UV-Visible spectrophotometer (Thermo Fisher Scientific Inc., Madison, WI, USA). The total flavonoid content (TFC) was determined based on the calibration curve (quercetin—QE). The results were expressed in mg QE/mL (mg quercetin equivalents/mL extract) and mg QE/g dry plant material (DW), respectively.

#### 2.4.4. Total Anthocyanins Content

The method proposed by Giusti and Wrolstad [46], named the pH variation method, is based on the reversible pH-dependent transformations that anthocyanin pigments undergo.

The total anthocyanins content (TAC) of grape pomace extracts was determined by measuring the absorbance of the solution at a single wavelength knowing that anthocyanins have a typical absorption band in the 490 ÷ 550 nm region of the visible spectrum. This band is separated from the absorption bands of other phenolic compounds, which have the maximum spectral in UV [47]. The applied procedure involved the following steps: (1) the dilution factor (DF) of the sample was determined by using a KCl 0.025 M buffer solution (pH = 1.0); therefore, the absorbance of the sample was less than 1.2; DF was set as the final volume of the sample divided by the initial volume of the extract; (2) the sample spectrum (260–800 nm; distilled water blank) was recorded to verify the position (*λ_max_*) and the amplitude (maximum absorbance value); *λ_max_* = 520 nm according to the method; (3) two dilutions of each extract sample were prepared, one with KCl 0.025 M buffer solution (*pH* = 1.0) and the second with CH_3_COONa 0.4 M buffer solution (*pH* = 4.5) and were maintained in equilibrium for 15 min; (4) the sample absorbance for the two dilutions was measured at 700 nm (*A*_700_) and at the one corresponding to the maximum absorption (*A_λmax_*) using an Evolution 260 Bio UV-Visible spectrophotometer (Thermo Fisher Scientific Inc., Madison, WI, USA). Finally, the absorbance of the diluted sample was calculated according to Equation (2):

(2) $$A={\left(A_{\lambda max}-A_{700}\right)}_{pH=1.0}-{\left(A_{\lambda max}-A_{700}\right)}_{pH=4.5}$$

The concentration of monomeric anthocyanin pigment (*MAP*), expressed as mg/L, was calculated with Equation (3):

(3) $$MAP=\frac{A\cdot MW\cdot DF\cdot 1000}{\varepsilon \cdot l}$$

where *A* is the absorbance of the diluted sample; *MW* is the molecular mass (g/mol); *DF* is the dilution factor; *ε* represents the molar absorptivity expressed as L/(mol·cm); *l* is the optical path length (1 cm, quartz cuvettes). *MW* and *ε* correspond to the predominant anthocyanin in the sample (*ε* value was taken from literature data for anthocyanin pigment in acidified aqueous solvent). According to the method, if *ε* for the majority pigment is not available, or if the composition of the sample is unknown, the pigment content must be calculated as cyanidin-3-glucoside, where *MW* = 449.2 g/mol and *ε* = 26,900 L/(mol·cm) [48].

#### 2.4.5. Total Condensed Tannins

The total condensed tannins (TCT) method is based on the reaction of flavanols (condensed tannins, monomers, dimers, etc.) which, unlike most natural phenolic compounds, react with vanillin in an acidic medium to form a colored compound that absorbs at 500 nm and gives the solution a red coloration that is quantified colorimetrically [49,50]. The procedure involved the following steps: (1) over 0.5 mL of the extracted sample was added to 3 mL of 2.4% vanillin solution, and the content was homogenized (vortex); (2) 1.5 mL of 30% hydrochloric acid was added to the mixture and homogenized again (vortex); (3) the mixture was held for 15 min at 20 ± 2 °C, then for 13 min at 25 °C. The blank was prepared similarly but with 0.5 mL of distilled water instead of extract. An interference correction sample (anthocyanins) was also prepared with 3 mL of HPLC-grade methanol (instead of vanillin solution). The absorbance was measured at 500 nm; the absorbance of the sample was calculated considering the contributions of the absorbance of the blank and the interference correction sample. The content of total condensed tannins (TCT) was determined based on the absorbance of the sample on the calibration curve, i.e., (+) catechin, expressed in catechin equivalents (CE); mg CE/g DW or mg CE/mL.

#### 2.4.6. High-Performance Liquid Chromatography

The identification and quantification of phenolic compounds in grape pomace extracts was performed using high-performance liquid chromatography with a 1260 Infinity II chromatograph (Agilent, Santa Clara, CA, USA) equipped with two detectors: a diode array detector (DAD) and a fluorescence detector (FLD). The obtained data were automatically processed with OpenLab CDS 2.7 software (Agilent Technologies Inc., Santa Clara, CA, USA). For a good separation and identification of polyphenols, chromatographic column type Agilent ZORBAX RRHD SB-C18 (Agilent Technologies Inc., Santa Clara, CA, USA) was used (pore size 80 Å; product size 100 × 2.1 mm; particle size 1.8 μm). The column temperature was set at 20 °C and the limit of quantification (LOQ) values of the method for standards, i.e., catechin, vanillic acid, caffeic acid, myricetin, resveratrol, and kaempferol were 0.015 ng/mL, 0.015 ng/mL, 0.014 ng/mL, 0.003 ng/mL, 0.021 ng/mL, and 0.016 ng/mL, respectively. The calibration curves of standard solutions (R^2^ > 0.999) were established in the range of 0.2–10.0 ppm (Tables S2–S6). The gradient elution program using solvent A (water H_2_O) and solvent B (acetonitrile CH_3_CN) was programmed as follows: 2% B (0–8 min), 17% B (8–14 min), 23% B (14–22 min), and 95% B linearly (22 min), with a 0.400 mL/min flow rate and a maximum pressure limit of 800 bars.

### 2.5. Statistical Analysis

Data were analyzed using IBM SPSS Statistics v. 26. A *t*-test, one-way ANOVA using the Duncan post-hoc test, Pearson’s correlation analysis, principal component analysis (PCA), and hierarchical cluster analysis (HA) were performed. A *p*-value < 0.05 was considered statistically significant.

## 3. Results and Discussion

### 3.1. Phytochemical Parameters Determined by UV-Vis Spectrometry

The phytochemical parameters of grape pomace extracts showed significant differences (*p* < 0.05) among the varieties of white and red grapes studied for TPC, TFC, TAC, TCT, and DPPH radical scavenging capacity (Table 1).

**Figure 2:**
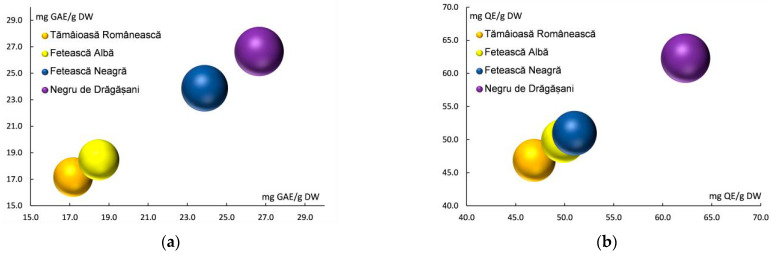
Total polyphenols content (**a**) and total flavonoid content (**b**) of grape pomace extracts of white and red varieties.

Figure 2 shows the total polyphenols content (a) and total flavonoid content (b) of grape pomace extracts for the four investigated grape varieties (Fetească Albă, Tămâioasă Românească, Fetească Neagră, and Negru de Drăgășani). Total polyphenols content (TPC) ranged between 17.161 ± 1.346 mg GAE/g DW (TR) and 26.654 ± 3.356 mg GAE/g DW (ND), emphasizing the higher values for red varieties relative to white ones (Table 1 and Figure 2a). The variation of total flavonoids content (TFC) was similar to the TPC variation, ranging between 46.875 ± 2.637 mg QE/g DW (TR) and 62.312 ± 4.670 mg QE/g DW (ND) (Table 1 and Figure 2b).

**Figure 3:**
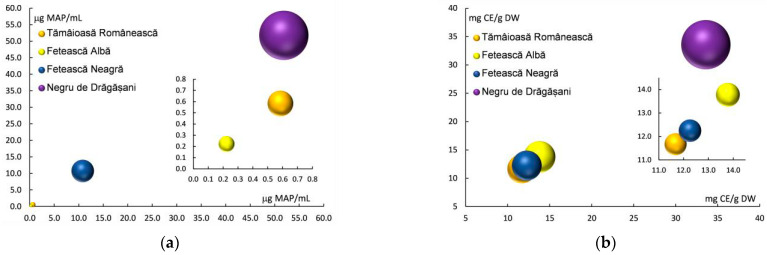
Total content of anthocyanins (**a**) and total condensed tannins (**b**) of grape pomace extracts of white and red varieties.

Figure 3 shows the total content of anthocyanins (a) and the total condensed tannins (b) of grape pomace extracts of the investigated grape varieties (Fetească Albă, Tămâioasă Românească, Fetească Neagră, and Negru de Drăgășani). The results obtained for total anthocyanins content (TAC) show significant differences between white and red varieties, ranging between 0.223 ± 0.048 μg MAP/mL (Fetească Albă) and 51.822 ± 4.668 μg MAP/mL (Negru de Drăgășani) (Table 1 and Figure 3a). The highest total condensed tannins (TCT) content was obtained for the Negru de Drăgășani red variety (33.575 ± 5.684 mg CE/g DW), while for the rest of the varieties, the values ranged from 11.671 ± 0.350 to 13.777 ± 1.710 mg CE/g DW.

**Figure 4:**
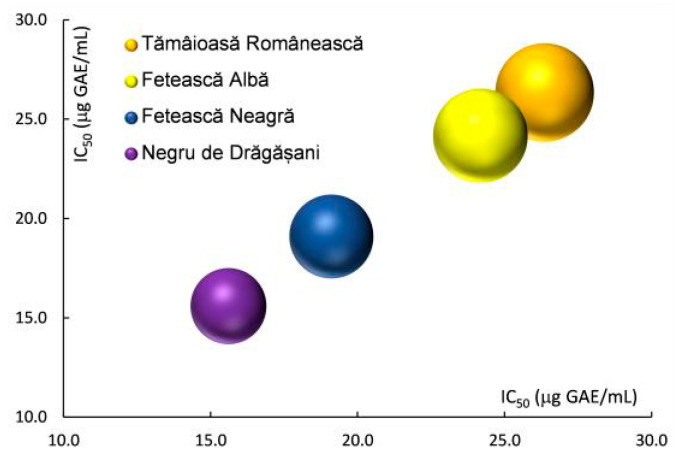
Half maximal inhibitory concentration (IC_50_)—DPPH assay of grape pomace extracts of white and red varieties.

Antioxidant activity (AA) expressed as half maximal inhibitory concentration (IC_50_) ranged from Negru de Drăgășani > Fetească Neagră > Fetească Albă > Tămâioasă Românească (Table 1 and Figure 4), suggesting a direct correlation with TPC and TFC.

### 3.2. Polyphenols Content Determined by High-Performance Liquid Chromatography

Chromatographic data/profile of the six polyphenols in the grape pomace extract of Fetească Albă, Tămâioasă Românească, Fetească Neagră, and Negru de Drăgășani varieties are presented in Tables S7–S10 and Figures S1–S4. The polyphenol content was correlated with TPC, TFC, TCA, TCT, and antioxidant activity using statistical analysis.

### 3.3. Discrimination of the White and Red Grape Pomaces

#### 3.3.1. Characterization of White and Red Grape Pomaces

A *t*-test was applied to determine whether there are significant differences between the groups of the extracts obtained from the pomace of the four grape varieties in terms of their phenolic compounds and antioxidant activity. The concentration of condensed tannins (TCT) and antioxidant activity (IC_50_) differed (*p* < 0.05) among Tâmâioasă Românească and Fetească Albă (white varieties). Total polyphenols content (TPC), total content of flavonoids (TFC), total anthocyanins (TAC), total condensed tannins (TAC), and antioxidant activity (IC_50_) differed (*p* < 0.05) among the red varieties (Negru de Dăgășani and Fetească Neagră).

According to Tukey’s HSD test, based on the means for groups in homogenous subsets (Table 2), the multiple comparisons reveal that the groups are different from each other in terms of the phytochemical parameters as follows:

- IC_50_ values: Tâmâioasă Românească, Fetească Albă, Fetească Neagră, and Negru de Dăgășani, respectively;
- TPC: the subset of the white grape varieties (Tâmâioasă Românească and Fetească Albă) differs significantly from the subset of the red varieties (Negru de Dăgășani and Fetească Neagră), respectively;
- TFC and TCT: the subset (Tâmâioasă Românească, Fetească Albă, and Fetească Neagră) is different from the subset of the red variety Negru de Dăgășani;
- TAC: there are three different subsets: Tâmâioasă Românească-Fetească Albă, Fetească Neagră, and Negru de Dăgășani, respectively.

**Figure 5:**
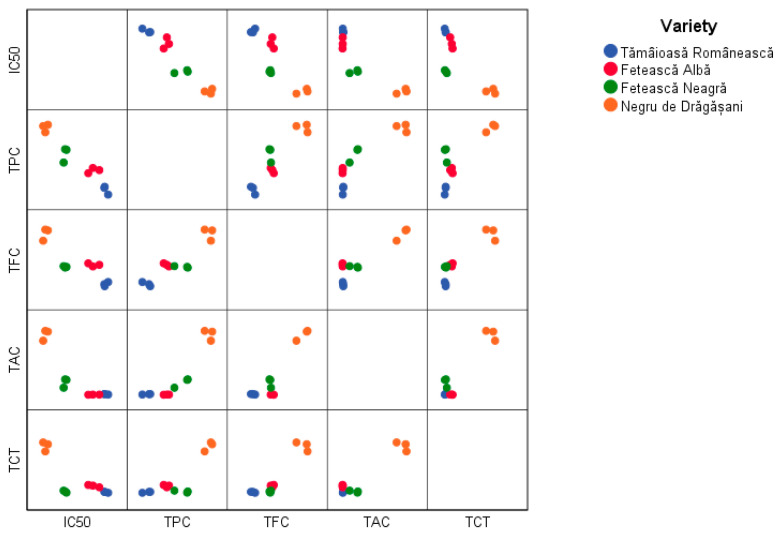
The matrix of IC_50_, TPC, TFC, TAC, and TCT of grape pomace extracts.

The matrix of the chemical parameters and the antioxidant activity for all varieties (Figure 5) showed that the pomace extract obtained from the Negru de Drăgășani variety was characterized by the lowest IC_50_ value, but also by the highest values of TPC, TFC, TAC, and TCT. Excepting TAC values, Fetească varieties occupied an intermediate position within the range of values determined for all experiments.

The investigated extracts obtained from grape pomace showed different concentrations of polyphenols and antioxidant activities, respectively. The TPC values of the four grape pomace extracts were slightly different from those determined by several authors who had applied different extraction methods to various ranges of grape varieties. If the black varieties are discussed, Negro et al. [51] reported phenolic content levels ranging between 52.9 (Negroamaro var.) and 27.6 (Malvasia di Lecce) mg/g DW. Negroamaro cv. showed the highest total flavonoid content (38.4 mg CE/g DW), while this parameter was equal to 20.7 mg CE/g DW for Malvasia di Lecce cv. Unlike our experiment, the grape pomace was extracted with methanol, but also in an ultrasonic bath. The TPC values are also influenced by several factors, such as the winemaking techniques and growing region [51].

Fetească Neagră pomace was subjected to two different extraction procedures [52]. The authors reported a total phenolic content of 2.775 ± 0.002 mg GAE/mL (extract with ethanol) and 1.452 ± 0.070 mg GAE/mL (extract with methanol). As can be observed, using different modalities for results quantification makes comparison of the data quite challenging. Four extraction procedures applied to the grape pomace obtained from the Fetească Neagră variety led to a total polyphenols concentration that ranged from 4 to 7 mg GAE/g DW [53].

A higher bleaching effect is associated with a lower IC_50_ value that represents, consequently, a better antioxidant activity [52]. The following order was established for antioxidant activity: Negru de Dăgășani > Fetească Neagră > Fetească Albă > Tâmâioasă Românească. The IC_50_ values observed in the Fetească Neagră grape pomace extract (19.103 ± 0.256 μg/mL) were similar to those determined by Luchian et al. [52] (i.e., 20.59 ± 0.75 μg/mL). Due to its important phenolic content, Fetească Neagră pomace extracts exhibited good in vitro antioxidant activity [54]. By discussing the antioxidant activity of phenolics, not only should their concentration be taken into account, but also the number of their hydroxyl groups.

The pomace from the Fetească Neagră cultivar was recommended as a source of polyphenols and several phenolic compounds [53] with potential application in the food industry or in food supplements. Its catechin content was reported as 50 mg/100 g pomace powder, the extraction method having an overwhelming influence at this level.

The profile of phenolic compounds in different pomace flours from four red hybrid grape cultivars grown in Brazil revealed that the TPC values ranged from 23.2 to 49.3 g/kg, while TAC was found at levels varying from 1.2 to 7.9 g/kg [55]. Anthocyanins were reported as the main phenolic compound in the majority of the grape pomace flour samples. This study revealed that Negru de Drăgășani had the greatest TAC, five times greater than that of Fetească Neagră.

The anthocyanin derivatives can vary greatly from sample to sample [51]. These have attracted a lot of interest recently in terms of nutraceuticals and benefits for human health, and further studies are needed to confirm these indications [51].

The data obtained in the current study related to the catechin fall within the averages of the values obtained by other authors. The concentration of this flavan-3-ol was reported to range within broad limits, from 0.02 to 1.50 mg/g; in addition, the role of climatic conditions and agronomic techniques in its amount is emphasized [51]. However, applying different sample preparations (briefly, the freeze-drying of pomace and extraction in aqueous acetone) [56] determined that the catechin concentration in the extracts of four Virginia-grown grape pomaces ranged from 910 ± 10.5 mg/g extract (Viognier cv.) to 214 ± 4.8 mg/g extract (Chambourcin cv.).

**Figure 6:**
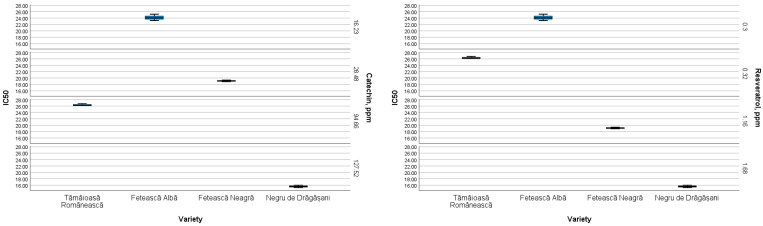

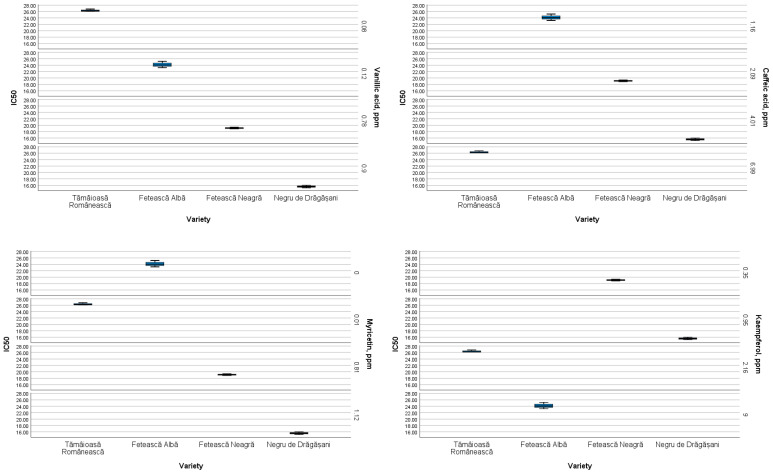
The matrices of IC_50_ of grape pomace extracts in relation to the phenolic compounds investigated by HPLC.

The boxplots (Figure 6) showed the existing relationship between antioxidant activity (expressed as IC_50_) and the concentration of each phenolic compound determined by HPLC, depending on the grape variety.

As can be observed, the phenolic compound concentration varied according to the grape cultivar. Thus, the catechin content ranged from 16.231 ppm in the Fetească Albă to 127.517 ppm in the Negru de Drăgășani pomace, which also contained a higher amount of resveratrol. A similar and lower quantity of resveratrol was determined in the pomaces obtained from the white varieties (Tămâioasă Românească and Fetească Albă, respectively). The most abundant compounds in these varieties were caffeic acid in Tămâioasă Românească and kaempferol in Fetească Albă. The other determined flavonol compound, namely myricetin, almost absent from the white grape cultivars, reached the highest levels in the red varieties (Fetească Neagră and Negru de Drăgășani).

The resveratrol content of Fetească Neagră pomace extracts was reported in amounts of 15.49 ± 0.09 μg/mL [52]. Interestingly, caffeic acid was reported as absent from the profile of phenolic compounds for all four types of extracts obtained from Fetească Neagră pomace [53]. The quantity of resveratrol identified in the extracts was lower than 5 mg/100 g grape pomace powder. In further studies, the quantification of all other representative polyphenolic compounds will be of great importance in better understanding the bioactive profile of grape pomace extracts.

#### 3.3.2. Pearson Analysis

Table 3 reports the correlation between the polyphenolic compounds determined in all four varieties’ samples and the results of the antioxidant activity. High levels of polyphenols and flavonoids were associated with high antioxidant activity.

The presented data show strong and inverse correlations (r > 0.95, *p* < 0.01) between IC_50_ and TPC, vanillic acid, myricetin, and resveratrol, respectively. The lowest statistically significant Pearson value within the group of data was determined between IC_50_ and TCT (r = −0.759, *p* < 0.01). However, this correlation is also strong (r > 0.75), as are those previously mentioned.

The antioxidant activity, expressed as IC_50_, was strong and inversely correlated with TAC, which is in agreement with the results reported by Lingua et al. [57]. According to these authors, anthocyanins are important for the antioxidant capacity of pomace. TPC showed a higher correlation with the antioxidant capacity (r = −0.960, *p* < 0.01) than the TAC (r = −0.868, *p* < 0.01), in line with the results reported by Monteiro et al. [55] for pomace flours of four red grape varieties.

A close relationship between phenolic compound content and antioxidant capacity was reported in the literature [51,52]. In this study, the TPC was positively correlated with TFC (r = 0.858, *p* < 0.01), TAC (r = 0.893, *p* < 0.01), and TCT (r = 0.744, *p* < 0.01), respectively. Our results are in line with those reported by Xu et al. [56], who investigated the pomace extracts from four Virginia-grown grape varieties. Total polyphenols, total flavonoids, and tannins exhibited significant, positive correlations with each other, as well as with the antioxidant activity. Interestingly, the lack of correlation was underlined between the above-mentioned compounds and TAC (although anthocyanins contribute to DPPH• antioxidant capacity), contrary to our results. The authors explained the capacity of each phenolic compound to scavenge different free radicals on the basis of its distinctive structure (particularly the number of OH groups, the side chain on benzoic acid).

A weak and non-statistically significant relationship was established between TPC and catechin content, as was observed by Onache et al. [58], for red varieties of must and wine obtained from grapes harvested at the Ștefănești Vineyard. Pearson’s correlation analysis applied to quantitative data revealed moderate correlations between catechin and caffeic acid (r = 0.731, *p* < 0.01), catechin and kaempferol (r = −0.548, *p* < 0.01), vanillic acid and kaempferol (r = −0.673, *p* < 0.01), myricetin and kaempferol (r = −0.685, *p* < 0.01), and resveratrol and kaempferol (r = −0.666, *p* < 0.01), respectively. Strong and moderate correlations between the main phenolic compounds found in musts and wines were also reported by Onache et al. [58].

#### 3.3.3. Regression Analysis

**Figure 7:**
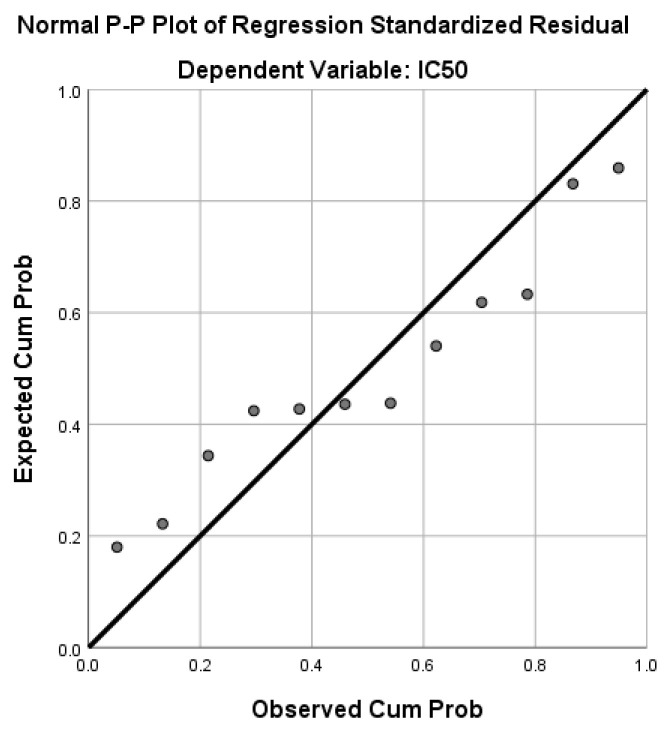
Normal P−P plot of regression standardized residual.

The relationship between the IC_50_ values corresponding to all pomace extracts and the other variables were evaluated and the results were interpreted through a multiple regression model. The experimental and predicted data for IC_50_ are shown in Figure 7. The regression model, which expresses the IC_50_ (μg GAE/mL) as a function of the seven experimental factors, is described by Equation (4).

(4) $$\begin{array}{cc} Pred\left({IC}_{50}\right)= & -44.27+1.164\cdot TPC+0.754\cdot TFC-0.443\cdot TAC-0.466\cdot TCT\\ & \;+0.040\cdot Catechin+2.591\cdot Caffeicacid+0.955\cdot Kaempferol \end{array}$$

Probably due to their cross effects, the grape variety, vanillic acid, myricetin, and resveratrol were excluded from the regression model, the R squared value of which was very close to 1 (R^2^ = 0.996). Taking into account the TFC amount and the determined phenolic compounds belonging to flavonoids, the TFC was also predicted with the help of multiple regressions performed in SPSS. Equation (5) describes the TFC (mg QE/g DW) as a function of the three flavonoids (R^2^ = 0.993).

(5) $$Pred\left(TFC\right)=42.665+0.294\cdot TAC+7.396\cdot Myricetin+1.138\cdot Kaempferol–0.008\cdot Catechin$$

**Figure 8:**
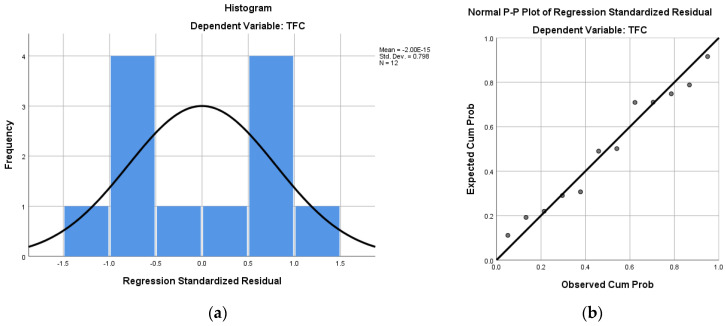
The histogram (**a**) and the normal P−P plot of regression standardized residual (**b**).

The graphical representations of the data distribution are shown in Figure 8a, while and the experimental and predicted data for TFC are shown in Figure 8b.

#### 3.3.4. Principal Component Analysis (PCA) and Hierarchical Cluster Analysis (HCA)

**Figure 9:**
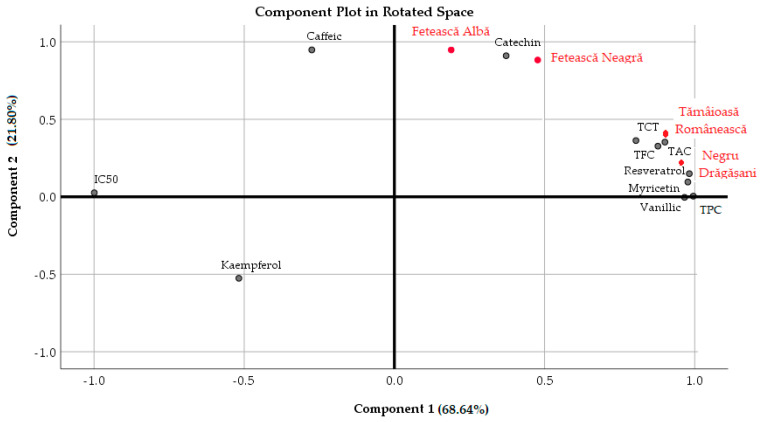
Principal component analysis of phenolic compounds (key variables) and the antioxidant capacity in the four pomace extracts.

Both PCA and HCA were performed in order to establish a descriptive model for grouping all four grape pomace samples based on their polyphenols content, phenolic compounds, and antioxidant activity (expressed as IC_50_). Principal component analysis summarizes the association between variables (TPC, TFC, TAC, TCT, IC_50_, and phenolic compounds) of the extracts obtained from the four grape varieties. The results of the factor analysis showed that PC1 and PC2 accounted for 90.44% of the data variance (Table 4, Figure 9).

PC1 explained a high percentage of the total variance. TPC, TFC, TAC, TCT, vanillic acid, myricetin, and resveratrol are located in its positive part, while IC_50_ is situated in the negative part of PC1 (in agreement with the results of the Pearson correlation). The structure of PC1 underscores the strong relationship between the phenolic content and the antioxidant activities of the grape pomace extracts. At the first iteration of the factor analysis, all four grape pomaces were grouped in the positive part of PC1. The rotated component matrix (rotation method—Varimax with Kaiser normalization) revealed the grouping of the grape varieties in two components on the basis of their chemical composition and not on their belonging to a variety of a certain color. Thus, Tămâioasă Românească and Negru de Drăgășani were clearly separated in PC1 (+), while Fetească Albă and Fetească Neagră were separated in PC2 (+). The interpretation of the first component loadings suggests that high TPC and TAC values and high vanillic acid, myricetin, and resveratrol concentrations concur with lower antioxidant activity (IC_50_ values) of the grape pomace extracts. As an indicator of free radical scavenging, IC_50_ showed the highest factor loading. The second component discriminates the grape pomace extracts depending on the composition of kaempferol, catechin, and caffeic acid. A similar separation of the caffeic acid (PC2 +) from kaempferol (PC2 −) was determined by Monteiro et al. [55] in a PCA of the antioxidant capacity and phenolic compounds in different grape pomace flours. In this study, PC2 is highly positively associated with catechin and caffeic acid and moderately negatively associated with kaempferol. These phenolic compounds were found to be uncorrelated with IC_50_, according to the above-mentioned Pearson values.

**Figure 10:**
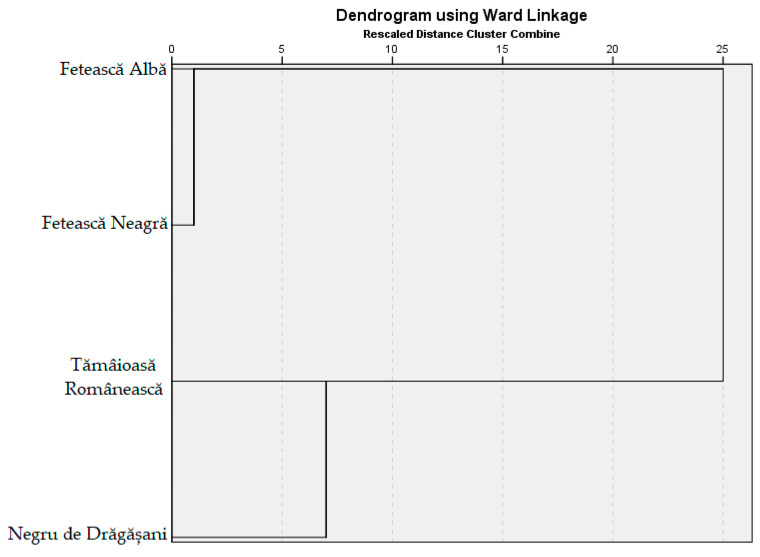
Dendrogram of variables of interest (Ward linkage method).

Hierarchical cluster analysis (HCA) was applied both on the experimental data (IC_50_, TPC, TFC, TAC, TCT, and phenolic compounds of the hydroalcoholic extracts of the grape pomace) and on the values of descriptors (white and red grapes varieties). The Ward linkage method and the squared Euclidean distance were used in clustering. The dendrogram associated with the analysis is shown in Figure 10. The affiliation of each grape pomace extract with a cluster in the first stage of clusterization was obtained as follows:

- Cluster 1: Fetească Albă and Fetească Neagră (two extracts);
- Cluster 2: Tămâioasă Românească and Negru de Drăgășani (two extracts).

The separation of Tămâioasă Românească and Negru de Drăgășani in Cluster 2 may be attributed to their levels of catechin and caffeic acid, which are distinct from the other two extracts. Extracts from Fetească Albă and Fetească Neagră were grouped in a distinct cluster, mainly due to similar values of TFC and TCT.

The HCA analysis, in accordance with PCA, highlighted that Tămâioasă Românească and Negru de Drăgășani, and Fetească Albă and Fetească Neagră, respectively, are similar to each other. The HCA grouped the extracts belonging to white and red grape varieties in different clusters, contrary to the results of Onache et al. [58], which reported the discrimination that occurs between white and red varieties in the case of musts and wines.

## 4. Conclusions

Based on the results of preliminary screening tests, the extraction of phenolic compounds from the pomace of Romanian white and red grape varieties using ethyl alcohol:water = 1:1 solvent and ultrasonication was the first step of this study. The significant differences among the pomace obtained from different grape varieties were determined in terms of their phenolic content. The total polyphenols content (TPC) and the total flavonoid content (TFC) of grape pomace extracts decreased as follows: Negru de Drăgășani > Fetească Neagră > Fetească Albă > Tămâioasă Românească. The red varieties (Fetească Neagră and Negru de Drăgășani) were the richest in the content of total phenolics and total anthocyanins, respectively. The presence of the different phenolics varied in relation to the grape variety. Thus, catechin was the most abundant phenolic compound determined in the pomace extract of the Negru de Drăgășani variety. The antioxidant activity of extracts obtained from grape pomace showed that Negru de Drăgășani had the higher antioxidant activity due to its high level of polyphenols, followed by the Fetească Neagră, Fetească Albă, and Tămâioasă Românească varieties, respectively. A high amount of bioactive polyphenolic substances, such as myricetin, resveratrol, and vanillic acid, was also determined in the pomace of the Negru de Drăgășani extract. The pomace from the Fetească Albă variety will be further investigated due to its higher amount of kaempferol, a compound with proven beneficial biological activities [51]. Overall, these results show that the pomace obtained from the four Romanian white and red grape varieties could be successfully valorized by its contribution to the sustainability of wine-making and innovation in the economic sectors where the added value of phenolic compounds is needed. The valorization of grape pomace has significant implications for food sustainability, with large applications in the cosmetic industry. Polyphenols from these grape residues can be used in the treatment of hypertension, diabetes, cancer, aging, neurodegenerative diseases, and obesity, contributing to the development of new food and cosmetic products.

## Figures and Tables

**Table 1:** Phytochemical parameters of grape pomace extracts from Fetească Albă (FA), Tămâioasă Românească (TR), Fetească Neagră (FN), and Negru de Drăgășani (ND) varieties.

Grape Variety	TPC [mg GAE/g DW]	TFC [mg QE/g DW]	TAC [μg MAP/mL]	TCT [mg CE/g DW]	IC_50_ [μg GAE/mL]
FA	18.472 ± 1.935 ^a^	49.833 ± 3.326 ^ab^	0.223 ± 0.048 ^a^	13.777 ± 1.710 ^a^	24.168 ± 0.993 ^a^
TR	17.161 ± 1.346 ^a^	46.875 ± 2.637 ^a^	0.584 ± 0.221 ^a^	11.671 ± 0.350 ^a^	26.350 ± 0.374 ^b^
FN	23.872 ± 1.172 ^b^	50.979 ± 1.094 ^b^	10.771 ± 3.982 ^b^	12.251 ± 0.723 ^a^	19.103 ± 0.256 ^c^
ND	26.654 ± 3.356 ^c^	62.312 ± 4.670 ^c^	51.822 ± 4.668 ^c^	33.575 ± 5.684 ^b^	15.598 ± 0.421 ^d^

The results are presented as mean (triplicate) ± standard deviation (SD). In the same column, different letters correspond to statistically different means (Duncan’s test, *p* < 0.05).

**Table 2:** Grouping the samples in homogenous subsets according to post-hoc analysis.

Parameter/Grape Variety	Tâmâioasă Românească	Fetească Albă	Fetească Neagră	Negru de Dăgășani
IC_50_	√	√	√	√
TPC	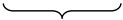		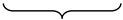	
TFC	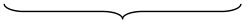			√
TAC	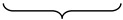		√	√
TCT	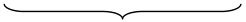			√

Note: associative grouping (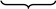); individual group (√).

**Table 3:** Pearson correlation analysis of the relationship between the antioxidant activity (expressed as IC_50_) and the polyphenolic compounds among the grape pomace extracts obtained from four varieties.

Parameter/ Compound	IC_50_	TPC	TFC	TAC	TCT	Catechin	Vanillic	Caffeic	Myricetin	Resveratrol	Kaempferol
IC_50_	1										
TPC	−**0.960** **	1									
TFC	−**0.862** **	**0.858** **	1								
TAC	−**0.868** **	**0.893** **	**0.919** **	1							
TCT	−**0.759** **	0.744 **	**0.913** **	**0.941** **	1						
Catechin	−0.341	0.318	0.524 **	0.698*	0.685 **	1					
Vanillic acid	−**0.966** **	**0.882** **	0.706 **	**0.798** **	0.626 **	0.309	1				
Caffeic acid	0.293	−0.227	−0.107	0.046	0.046	0.731 **	−0.228	1			
Myricetin	−**0.973** **	**0.886** **	**0.754** **	**0.859** **	0.700 **	0.415 *	**0.993** **	−0.148	1		
Resveratrol	−**0.974** **	**0.884** **	**0.791** **	**0.901** **	**0.755** **	0.480 *	**0.978** **	−0.110	**0.996** **	1	
Kaempferol	0.519	−0.523 **	−0.302	−0.478	−0.278	−0.548 **	−0.673 **	−0.464 *	−0.685 **	−0.666 **	1

* Correlation is significant at the 0.05 level (2-tailed). ** Correlation is significant at the 0.01 level (2-tailed). The strong correlations were mentioned in Bold (Pearson values from 1.0 to 0.75).

**Table 4:** Factor loadings (Varimax normalized) using principal component extraction.

Factor	Component 1	Component 2
Eigenvalue	7.93	2.01
Cumulative variance (%)	72.14	90.44
IC_50_	−0.999	0.026
TPC	0.995	0.004
TFC	0.877	0.327
TAC	0.901	0.353
TCT	0.804	0.363
Catechin	0.372	0.91
Vanillic acid	0.966	−0.004
Caffeic acid	−0.275	0.948
Myricetin	0.977	0.095
Resveratrol	0.982	0.149
Kaempferol	−0.518	−0.526

## Data Availability

All research data are reported in this manuscript and in the Supplementary Materials.

## Supplementary Materials

The following supporting information can be downloaded at: https://www.mdpi.com/article/10.3390/antiox13091133/s1, Table S1. Standard polyphenols used for stock solutions for calibration; Table S2. Calibration data of standard polyphenols (0.2 ppm)—HPLC-DAD-FLD; Table S3. Calibration data of standard polyphenols (1.0 ppm)—HPLC-DAD-FLD; Table S4. Calibration data of standard polyphenols (2.5 ppm)—HPLC-DAD-FLD; Table S5. Calibration data of standard polyphenols (5 ppm)—HPLC-DAD-FLD; Table S6. Calibration data of standard polyphenols (10 ppm)—HPLC-DAD-FLD; Table S7. Chromatographic data of the six polyphenols in the grape pomace extract of Fetească Albă; Figure S1. Chromatographic profile of the six polyphenols in the grape pomace extract of Fetească Albă: (a) (+)catechin; (b) vanillic acid; (c) caffeic acid; (d) myricetin; (e) resveratrol; (f) kaempferol; Table S8. Chromatographic data of the six polyphenols in the grape pomace extract of Tămâioasă Românească; Figure S2. Chromatographic profile of the six polyphenols in the grape pomace extract of Tămâioasă Românească: (a) (+)catechin; (b) vanillic acid; (c) caffeic acid; (d) myricetin; (e) resveratrol; (f) kaempferol; Table S9. Chromatographic data of the six polyphenols in the grape pomace extract of Fetească Neagră; Figure S3. Chromatographic profile of the six polyphenols in the grape pomace extract of Fetească Neagră: (a) (+)catechin; (b) vanillic acid; (c) caffeic acid; (d) myricetin; (e) resveratrol; (f) kaempferol; Table S10. Chromatographic data of the six polyphenols in the grape pomace extract of Negru de Drăgășani; Figure S4. Chromatographic profile of the six polyphenols in the grape pomace extract of Negru de Drăgășani: (a) (+)catechin; (b) vanillic acid; (c) caffeic acid; (d) myricetin; (e) resveratrol; (f) kaempferol.

## References

[1] International Organisation of Vine and Wine (OIV). https://www.oiv.int/public/medias/7909/oiv-state-of-the-world-vitivinicultural-sector-in-2020.pdf.

[2] Lugo S.D.R., Kimita K., Nishino N. (2023). Characteristics of decision process towards circular food economy: A review. Clean. Logist. Supply Chain.

[3] Lavelli V. (2021). Circular food supply chains–Impact on value addition and safety. Trends Food Sci. Technol..

[4] Beres C., Costa G.N.S., Cabezundo I., da Silva-James N.K., Teles A.S.C., Cruz A.P.G., Mellinger-Silva C., Tonon R.V., Cabral L.M.C., Freitas S.P. (2017). Towards integral utilization of grape pomace from winemaking process: A review. Waste Manag..

[5] Lavelli V., Kerr W.L., García-Lomillo J., González-San José M.L., Galanakis C.M. (2017). Applications of recovered bioactive compounds in food products. Handbook of Grape Processing By-Products: Sustainable Solutions.

[6] Genisheva Z., Soares M., Oliveira J.M., Carvalho J., Saleh H.M., Hassan A.I., Aglan R.F. (2024). Wine Production Wastes, Valorization, and Perspectives. Advances and Challenges in Hazardous Waste Management.

[7] Cotoras M., Vivanco H., Melo R., Aguirre M., Silva E., Mendoza L. (2014). In Vitro and in Vivo Evaluation of the Antioxidant and Prooxidant Activity of Phenolic Compounds Obtained from Grape (*Vitis vinifera*) Pomace. Molecules.

[8] Ky I., Crozier A., Cros G., Teissedre P.-I. (2014). Polyphenols composition of wine and grape sub-products and potential effects on chronic diseases. J. Nutr. Health Aging.

[9] Ky I., Lorrain B., Kolbas N., Crozier A., Teissedre P.-L. (2014). Wine by-products: Phenolic Characterization and Antioxidant Activity Evaluation of Grapes and Grape Pomaces from Six Different French Grape Varieties. Molecules.

[10] Pereira P., Palma C., Ferreira-Pêgo C., Amaral O., Amaral A., Rijo P., Gregório J., Palma L., Nicolai M. (2020). Grape pomace: A potential ingredient for the human diet. Foods.

[11] Radulescu C., Buruleanu L.C., Nicolescu C.M., Olteanu R.L., Bumbac M., Holban G.C., Simal-Gandara J. (2020). Phytochemical Profiles, Antioxidant and Antibacterial Activities of Grape (*Vitis vinifera* L.) Seeds and Skin from Organic and Conventional Vineyards. Plants.

[12] Topalovic A., Knezevic M., Bajagic B., Ivanovic L., Milasevic I., Djurovic D., Mugosa B., Podolski-Renic A., Pesic M. (2020). Grape (*Vitis vinifera* L.): Health benefits and effects of growing conditions on quality parameters. Biodiversity and Biomedicine. Our Future.

[13] Torre E., Iviglia G., Cassinelli C., Morra M., Russo N. (2020). Polyphenols from grape pomace induce osteogenic differentiation in mesenchymal stem cells. Int. J. Mol. Med..

[14] Xia L., Xu C., Huang K., Lu J., Zhang Y. (2019). Evaluation of phenolic compounds, antioxidant and antiproliferative activities of 31 grape cultivars with different genotypes. J. Food Biochem..

[15] Yang J., Xiao Y.Y. (2013). Grape Phytochemicals and Associated Health Benefits. Crit. Rev. Food Sci. Nutr..

[16] Sinrod A.J.G., Shah I.M., Surek E., Barile D. (2023). Uncovering the promising role of grape pomace as a modulator of the gut microbiome: An in-depth review. Heliyon.

[17] Kato-Schwaetz C.G., Gomez Correa R.C., de Souza Lima D., De Sa-Nakanishi A.B., de Almeida Goncalves G., Seixas F.A.V., Haminiuk C.W.I., Barros L., Ferreira I.C.F.R., Bracht A. (2020). Potential anti-diabetic properties of Merlot grape pomace extract: An in vitro, in silico and in vivo study of α-amylase and α-glucosidase inhibition. Food Res. Int..

[18] Machado T.O.X., Portugal I., Kodel H.A.C., Droppa-Almeida D., Dos Santos Lima M., Fathi F., Oliveira M.B.P.P., de Albuquerque-Junior R.L.C., Dariva C., Souto E.B. (2024). Therapeutic potential of grape pomace extracts: A review of scientific evidence. Food Biosci..

[19] Goncalves G.A., Soares A.A., Correa R.C.G., Barros L., HaMinuk C.W.I., Peralta R.M., Ferreira I.C.F.R., Bracht A. (2017). Merlot grape pomace hydroalcoholic extract improves the oxidative and inflammatory states of rats with adjuvant-induced arthritis. J. Funct. Foods.

[20] Milinčić D.D., Stanisavljević N.S., Kostić A.Ž., Bajić S.S., Kojić M.O., Gašić U.M., Barać M.B., Stanojević S.P., Tešić Ž.L., Pešić M.B. (2021). Phenolic compounds and biopotential of grape pomace extracts from Prokupac red grape variety. LWT.

[21] Elejalde E., Villarán M.A., Lopez-de-Armentia I., Ramón D., Murillo R., Alonso R.M. (2022). Study of Unpicked Grapes Valorization: A Natural Source of Polyphenolic Compounds and Evaluation of Their Antioxidant Capacity. Resources.

[22] Radulescu C., Buruleanu L.C., Olteanu R.L., Nicolescu C.M., Bumbac M., Gorghiu L.M., Nechifor (Tudorache) M.D., Barros A.N. (2023). Grape by-products: Potential sources of phenolic compounds for novel functional foods. Functional Food-Upgrading Natural and Synthetic Sources.

[23] Ferrer-Gallego R., Silva P. (2022). The Wine Industry By-Products: Applications for Food Industry and Health Benefits. Antioxidants.

[24] Nicolescu C.M., Bumbac M., Radulescu C., Buruleanu L.C., Olteanu R.L., Gorghiu L.M., Teodorescu G., Holban G.C., Morata A., Loira I., González C. (2022). Romanian Organic and Conventional Red Grapes Vineyards as Potential Sources of High Value-Added Products. In Circular Economy Approach. Grapes and Wine.

[25] Pedrali D., Barbarito S., Lavelli V. (2020). Encapsulation of grape seed phenolics from winemaking byproducts in hydrogel microbeads–Impact of food matrix and processing on the inhibitory activity towards α-glucosidase. LWT.

[26] Tremlova B., Havlova L., Benes P., Zemancova J., Buchtova H., Tesikova K., Dordevic S., Dordevic D. (2022). Vegetarian “Sausage Ferrer s” with the Addition of Grape Flour. Appl. Sci..

[27] Iuga M., Mironeasa S. (2020). Potential of grape byproducts as functional ingredients in baked goods and pasta. Compr. Rev. Food Sci. Food Saf..

[28] García-Lomillo J., González-Sanjosé M.L., Del Pino-García R., Rivero-Pérez M.D., Muñiz-Rodríguez P. (2014). Antioxidant and antimicrobial properties of wine byproducts and their potential uses in the food industry. J. Agric. Food Chem..

[29] Gerardi C., D’Amico L., Migoni D., Santino A., Salomone A., Carluccio M.A., Giovinazzo G. (2020). Strategies for reuse of skins separated from grape pomace as ingredient of functional beverages. Front. Bioeng. Biotechnol..

[30] de la Cerda-Carrasco A., López-Solís R., Nuñez-Kalasic H., Peña-Neira Á., Obreque-Slier E. (2015). Phenolic composition and antioxidant capacity of pomaces from four grape varieties (*Vitis vinifera* L.). J. Sci. Food Agric..

[31] Cotea V.D., Barbu N., Grigorescu C., Cotea V.V. (2010). Podgoriile și Vinurile României.

[32] Goldammer T. (2018). Grape Grower’s Handbook. A Guide to Viticulture for Wine Production.

[33] REVINO Gourmet Drumul Vinului in Podgoria Dealu Mare–Oenoturism. https://revino.ro/podgoria-dealu-mare-urlati-ceptura-mizil-tohani-pietroasele-sahateni-drumul-vinului-a362.html.

[34] Jemrić T. A Guide to Harvesting and Maintaining the Quality of Table Grapes. https://seerural.org/wp-content/uploads/2024/03/A-guide-to-harvesting-and-maintaining-the-quality-of-table-grapes.pdf.

[35] Lacombe T., Audeguin L., Boselli M., Bucchetti B., Cabello F., Chatelet P., Crespan M., D’Onofrio C., Eiras Dias J., Ercisli S. *Grapevine European towards a Comprehensive List, European Project GrapeGen 06-Grapevine Genetic Resources*; Version 18 Pomaceh 2011, Annex 1 A: List of the Grape Varieties Registered in the Member States of the European Union, Annex 3 A: Official National Catalogs of Grape Varieties for Member States of the European Union, 2011. http://www.eu-vitis.de/docs/eucatgrape/ANNEX_1A_V3-4.pdf.

[36] Priewe J. (2015). Vinul de la Strugure în Pahar, Tot ce Trebuie să știm Despre Vin.

[37] Nicolescu C.M., Bumbac M., Radulescu C., Buruleanu L.C., Olteanu R.L., Stanescu S.G., Gorghiu L.M., Serban S.B., Buiu O. (2023). Phytochemical statistical mapping of red grapes varieties cultivated in Romanian organic and conventional vineyards. Plants.

[38] Radulescu C., Nicolescu M.C., Olteanu R.L., Bumbac M., Buruleanu L.C., Gorghiu L.M., Holban G.C. (2022). Dry Skin Emollient Cream with Skin/Seed Extract (*Vitis vinifera* L., Fetească Neagră Variety). Patent.

[39] Radulescu C., Olteanu R.L., Nicolescu C.M., Bumbac M., Buruleanu L., Holban C.G. (2021). Vibrational Spectroscopy Combined with Chemometrics as Tool for Discriminating Organic vs. Conventional Culture Systems for Red Grape Extracts. Foods.

[40] Nicolescu C.M., Bumbac M., Olteanu R.L., Alecu G.C., Boboacă-Mihaescu D.N., Necula C., Radulescu C. (2019). Influence of extraction method on chemical composition from red grapes skin extract. J. Sci. Arts.

[41] Radulescu C., Olteanu R.L., Sihi C., Florescu M., Lazurca D., Dulama I.D., Stirbescu R.M., Teodorescu S. (2019). Chemometric Assessment of Spectroscopic Techniques and Antioxidant Activity for *Hippophae rhamnoides* L. Extracts Obtained by Different Isolation Methods. Anal. Lett..

[42] Shimamura T., Sumikura Y., Yamazaki T., Tada A., Kashiwagi T., Ishikawa H., Matsui T., Sugimoto N., Akiyama H., Ukeda H. (2014). Applicability of the DPPH assay for evaluating the antioxidant capacity of food additives-inter-laboratory evaluation study. Anal. Sci..

[43] Sebaugh J.L. (2011). Guidelines for accurate EC50/IC50 estimation. Pharm. Statist..

[44] (2005). Determination of Substances Characteristic of Green and Black Tea/Part 1: Content of Total Polyphenols in Tea-Colorimetric Method Using Folin-Ciocalteu Reagent.

[45] Kamtekar S., Keer V., Patil V. (2014). Estimation of Phenolic content, Flavonoid content, Antioxidant and Alpha-amylase Inhibitory Activity of Marketed Polyherbal Formulation. J. Appl. Pharm. Sci..

[46] Giusti M.M., Wrolstad R.E. (2001). Characterization and measurement of anthocyanins by UV-visible spectroscopy. Curr. Protoc. Food Anal. Chem..

[47] Fuleki T., Francis F.J. (1968). Quantitative Methods for Anthocyanins. 4. Determination of Individual Anthocyanins in Cranberry and Cranberry Products. J. Food Sci..

[48] Jurd L., Asen S. (1966). The formation of metal and “co-pigment” complexes of cyanidin 3-glucoside. Phytochemistry.

[49] Broadhurst R.B., Jones W.T. (1978). Analysis of Condensed Tannins Using Acidified Vanilin. J. Sci. Food Agric..

[50] Rebaya A., Belghith S.I., Baghdikian B., Leddet V.M., Mabrouki F., Olivier E., Cherif J.K., Ayadi M.T. (2014). Total Phenolic, Total Flavonoid, Tannin Content, and Antioxidant Capacity of *Halimium halimifolium* (Cistaceae). J. Appl. Pharm. Sci..

[51] Negro C., Aprile A., Luvisi A., De Bellis L., Miceli A. (2021). Antioxidant Activity and Polyphenols Characterization of Four Monovarietal Grape Pomaces from Salento (Apulia, Italy). Antioxidants.

[52] Luchian C.E., Cotea V.V., Vlase L., Toiu A.M., Colibaba L.C., Raschip I.E., Nadaş G., Gheldiu A.M., Tuchilus C., Rotaru L. (2019). Antioxidant and antimicrobial effects of grape pomace extracts. BIO Web Conf..

[53] Frum A., Georgescu C., Gligor F.G., Lengyel E., Stegarus D.I., Dobrea C.M., Tita O. (2018). Identification and quantification of phenolic compounds from red grape pomace. Sci. Study Res. Chem. C.

[54] Balea S.S., Parvu A.E., Pop N., Marin F.Z., Parvu M. (2018). Polyphenolic Compounds, Antioxidant, and Cardioprotective Effects of Pomace Extracts from Fetească Neagră Cultivar. Oxid. Med. Cell. Longev..

[55] Monteiro G.C., Minatel I.O., Pimentel A., Gomez-Gomez H.A., Corrêa de Camargo J.P., Diamante M.S., Pereira Basílio L.S., Tecchio M.A., Pereira Lima G.P. (2020). Bioactive compounds and antioxidant capacity of grape pomace flours. LWT.

[56] Xu Y., Burton S., Kim C., Sismour E. (2015). Phenolic compounds, antioxidant, and antibacterial properties of pomace extracts from four Virginia-grown grape varieties. Food Sci. Nutr..

[57] Lingua M.S., Fabani M.P., Wunderlin D.A., Baroni M.V. (2016). From grape to wine: Changes in phenolic composition and its influence on antioxidant activity. Food Chem..

[58] Onache P.A., Florea A., Geana E.I., Ciucure C.T., Ionete R.E., Sumedrea D.I., Tita O. (2023). Assessment of Bioactive Phenolic Compounds in Musts and the Corresponding Wines of White and Red Grape Varieties. Appl. Sci..

