# Supplementary material for: Polyphenolic Screening and the Antioxidant Activity of Grape Pomace Extracts of Romanian White and Red Grape Varieties
Source: Antioxidants (Basel). 2024 Sep 19;13(9):1133. doi: 10.3390/antiox13091133 (PMC11429185; doi:10.3390/antiox13091133)
Supplement: Supplementary file 1 [file antioxidants-13-01133-s001.zip › antioxidants-3209369-supplementary.pdf]

Supplementary Material

Polyphenolic Screening and the Antioxidant Activity of Grape Pomace Extracts of Romanian White and Red Grape Varieties

Cristiana Radulescu[1,2,3*], Radu-Lucian Olteanu[1*], Lavinia-Claudia Buruleanu[4*], Mihaela Nechifor (Tudorache)[2], Ioana-Daniela Dulama[5], Raluca-Maria Stirbescu[5], Ioan-Alin Bucurica[5], Sorina-Geanina Stanescu[5], Andreea-Laura Banica[2,5]

1 Faculty of Sciences and Arts, Valahia University of Targoviste, 13 Sinaia Alley, 130004 Targoviste, Romania
2 Doctoral School Chemical Engineering and Biotechnology, National University of Science and Technology Politehnica of Bucharest, 313 Splaiul Independentei, 060042 Bucharest, Romania; tudorache.mihaela-db@ansvsa.ro (M.N.); banica.andreea@icstm.ro (A.-L.B.)
3 Academy of Romanian Scientists, 3 Ilfov, 050044 Bucharest, Romania
4 Faculty of Environmental Engineering and Food Science, Valahia University of Targoviste, 13 Sinaia Alley, 130004 Targoviste, Romania
5 Institute of Multidisciplinary Research for Science and Technology, Valahia University of Targoviste, 13 Sinaia Alley, 130004 Targoviste, Romania; dulama.ioana@icstm.ro (I.-D.D.); stirbescu.raluca@icstm.ro (R.-M.S.); geanina.stanescu@icstm.ro (S.-G.S.); bucurica_alin@icstm.ro (I.-A.B.)
* Correspondence: cristiana.radulescu@valahia.ro (C.R.); radu.olteanu@valahia.ro (R.-L.O.); lavinia.buruleanu@valahia.ro (C.-L.B.)

Polyphenols content determined by high-performance liquid chromatography

Grape pomace samples for high-performance liquid chromatography (HPLC) were prepared by ultrasonication. HPLC method was developed and optimized for detection and separation of six polyphenols (i.e., catechin, vanillic acid, caffeic acid, myricetin, resveratrol, and kampherol).

The calibration solutions 0.1 - 5.0 ppm were prepared by successive dilutions from a common stock containing the six polyphenols with a concentration of 100 ppm in methanol. The exact concentrations are represented in the Table S1. Volumes ranged between 50 – 200 μL were aliquoted from individual stocks and then brought to 1000 μL with methanol.

The calibration curves of standard solutions (R^2 > 0.999) were established in the range of 0.2 – 10.0 ppm (Table S2-S6).

Chromatographic data/profile of the six polyphenols in the grape pomace extract of Fetească Albă, Tămâioasă Românească, Fetească Neagră, and Negru de Drăgășani varieties are presented in Tables S7-S10 and Figures S1 – S4.

Table S1. Standard polyphenols used for stock solutions for calibration.

No	Polyphenols	Weight [mg]	Concentration [ppm]	Volume [µL]
1.	(+)Catechin	12.5	1250	100
2.	Caffeic acid	21.6	2160	50
3.	Vanillic acid	22.4	2240	50
4.	Kaempferol	5.0	500	200
5.	Myricetin	4.1	410	200
6.	Resveratrol	13.2	1320	100

Table S2. Calibration data of standard polyphenols (0.2 ppm) – HPLC-DAD-FLD

Name	RT [min]	Area	RF	Calibration Amount [ppm]	Amount [ppm]	Concentration [mg/kg]
(+)Catechin	12.216	0.677	2.286	0.250	0.296	0.2959
Vanillic acid	13.168	1.954	7.562	0.224	0.258	0.2584
Caffeic acid	13.390	7.511	31.659	0.216	0.237	0.2373
Myricetin	19.663	3.123	18.163	0.164	0.172	0.1719
Resveratrol	20.360	11.106	39.020	0.262	0.285	0.2846
Kaempferol	22.250	5.114	23.058	0.200	0.222	0.2218

Table S3. Calibration data of standard polyphenols (1.0 ppm) – HPLC-DAD-FLD

Name	RT [min]	Area	RF	Calibration Amount [ppm]	Amount [ppm]	Concentration [mg/kg]
(+)Catechin	12.207	2.744	2.376	1.250	1.155	1.1546
Vanillic acid	13.156	8.443	7.925	1.120	1.065	1.0654
Caffeic acid	13.377	32.336	30.318	1.080	1.067	1.0665
Myricetin	19.656	14.408	17.939	0.820	0.803	0.8032
Resveratrol	20.355	47.462	36.931	1.310	1.285	1.2852
Kaempferol	22.247	21.366	21.820	1.000	0.979	0.9792

Table S4. Calibration data of standard polyphenols (2.5 ppm) – HPLC-DAD-FLD

Name	RT [min]	Area	RF	Calibration Amount [ppm]	Amount [ppm]	Concentration [mg/kg]
(+)Catechin	12.205	7.511	2.396	3.125	3.135	3.1347
Vanillic acid	13.152	22.571	7.997	2.800	2.822	2.8224
Caffeic acid	13.373	80.756	30.087	2.700	2.684	2.6841
Myricetin	19.656	36.526	17.902	2.050	2.040	2.0404
Resveratrol	20.355	119.150	36.571	3.275	3.258	3.2581
Kaempferol	22.245	53.856	21.600	2.500	2.493	2.4934

Table S5. Calibration data of standard polyphenols (5 ppm) – HPLC-DAD-FLD

Name	RT [min]	Area	RF	Calibration Amount [ppm]	Amount [ppm]	Concentration [mg/kg]
(+)Catechin	12.196	15.101	2.402	6.250	6.287	6.2873
Vanillic acid	13.142	44.637	8.019	5.600	5.566	5.5664
Caffeic acid	13.362	161.601	30.011	5.400	5.385	5.3848
Myricetin	19.655	73.397	17.889	4.100	4.103	4.1028
Resveratrol	20.355	238.293	36.453	6.550	6.537	6.5370
Kaempferol	22.245	107.600	21.528	5.000	4.998	4.9981

Table S6. Calibration data of standard polyphenols (10 ppm) – HPLC-DAD-FLD

Name	RT [min]	Area	RF	Calibration Amount [ppm]	Amount [ppm]	Concentration [mg/kg]
(+)Catechin	12.194	30.029	2.405	12.500	12.488	12.4876
Vanillic acid	13.140	90.065	8.030	11.200	11.216	11.2160
Caffeic acid	13.359	324.071	29.973	10.800	10.812	10.8123
Myricetin	19.653	146.685	17.883	8.200	8.202	8.2023
Resveratrol	20.351	477.217	36.394	13.100	13.112	13.1124
Kaempferol	22.244	215.014	21.493	10.000	10.004	10.0040

Table S7. Chromatographic data of the six polyphenols in the grape pomace extract of Fetească Albă

No	Polyphenol	Detector	RT [min]	Area	Area 100%	Height	Height 100%	Amount [ppm]	Concentration [mg/kg]	S/N	Symmetry	Tailing	Plates EP	Width 50%[min]
1.	(+)Catechin	DAD	12.131	37.74	100.000	13.9	100.00	15.692	156.917	59	0.90	0.92	401847	0.045
2.	Vanillic acid	FLD	13.068	0.79	100.000	0.2	100.00	0.113	1.134	15	1.06	1.08	147716	0.080
3.	Caffeic acid	DAD	13.205	35.42	100.000	6.9	100.00	1.170	11.695	72	0.89	1.03	170641	0.075
4.	Myricetin	DAD	20.316	11.75	100.000	3.5	100.00	0.302	3.023	31	0.93	0.87	839206	0.052
5.	Resveratrol	DAD	22.216	196.58	91.194	58.9	91.52	9.145	91.449	332	0.93	1.08	1057063	0.051
6.	Kaempferol	DAD	12.131	37.74	100.000	13.9	100.00	15.692	156.917	59	0.90	0.92	401847	0.045

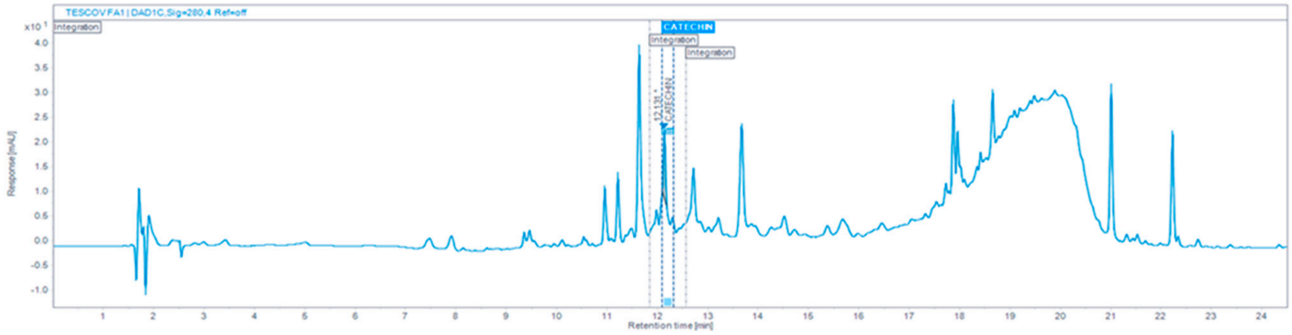

a

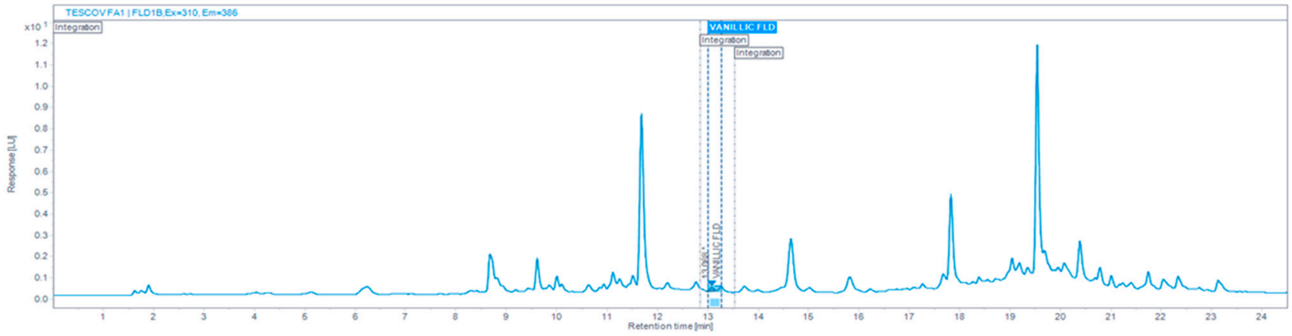
TESCOVFA1 | FLD1B,Ex=310, Em=386
VANILLIC FLD
Integration
Response [LU]
Retention time [min]

b

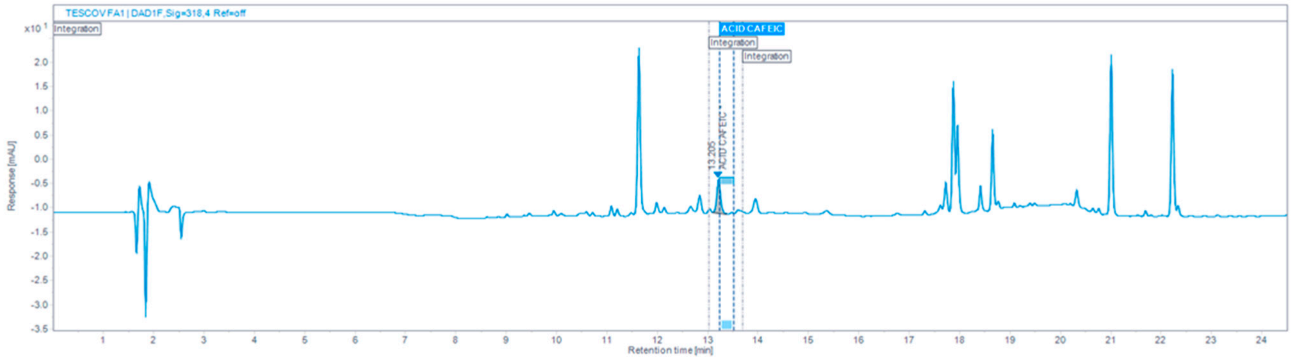
TESCOVFA1 | DAD1F,Sig=318,4 Ref=off
ACID CAFEIC
Integration
13.205
Response [mAU]
Retention time [min]

c

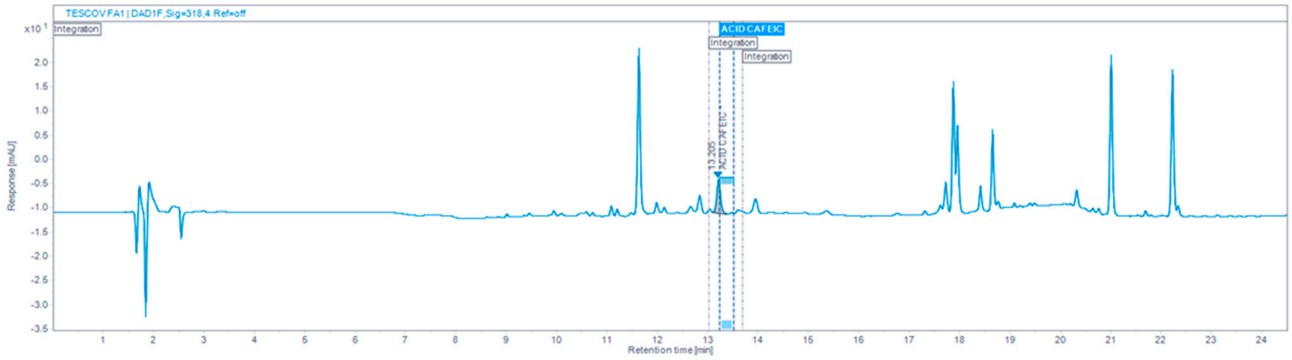
TESCOVFA1 | DAD1F,Sig=318,4 Ref=off
ACID CAFEIC
Integration
13.205
Response [mAU]
Retention time [min]

d

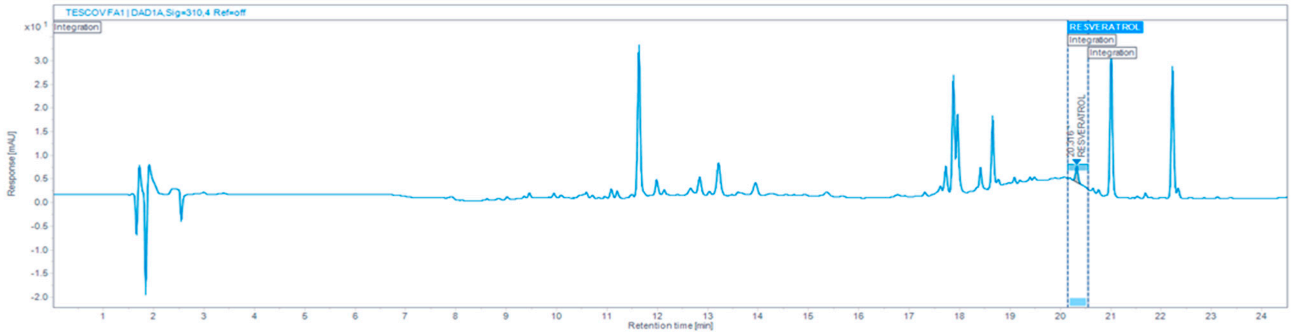
TESCOVFA1 | DAD1A,Sig=310,4 Ref=off
RESVERATROL
Integration
Response [mAU]
Retention time [min]

e

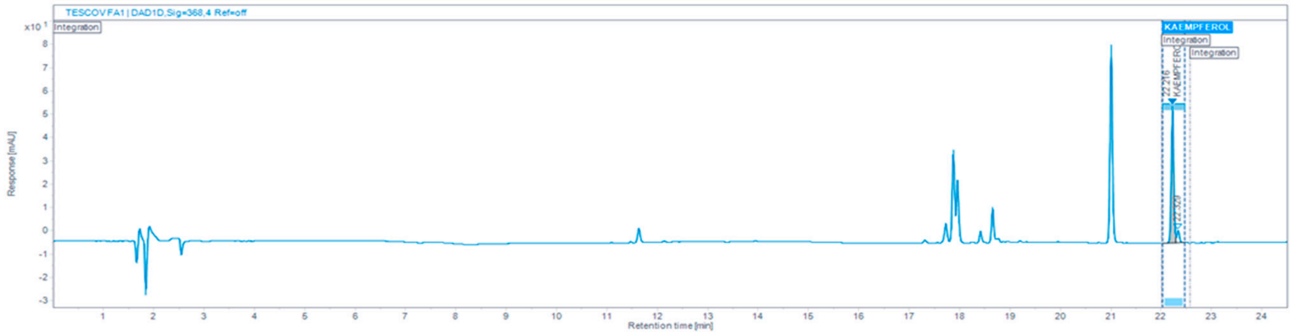

f

Figure S1. Chromatographic profile of the six polyphenols in the grape pomace extract of Fetească Albă: (a) (+)catechin; (b) vanillic acid; (c) caffeic acid; (d) myricetin; (e) resveratrol; (f) kaempferol.

Table S8. Chromatographic data of the six polyphenols in the grape pomace extract of Tămâioasă Românească

No	Polyphenol	Detector	RT [min]	Area	Area 100%	Height	Height 100%	Amount [ppm]	Concentration [mg/kg]	S/N	Symmetry	Tailing	Plates EP	Width 50%[min]
1.	(+)Catechin	DAD	12.144	241.99	100.000	70.8	100.00	100.528	1005.285	375	0.97	0.95	286691	0.053
2.	Vanillic acid	FLD	13.099	0.51	100.000	0.1	100.00	0.079	0.792	11	0.86	0.88	157033	0.078
3.	Caffeic acid	DAD	13.227	203.50	100.000	42.4	100.00	6.784	67.844	260	0.99	0.97	185082	0.072
4.	Myricetin	DAD	19.619	0.25	100.000	0.1	100.00	0.011	0.110	0	0.91	1.02	1035768	0.045
5.	Resveratrol	DAD	20.324	12.27	100.000	3.5	100.00	0.317	3.166	20	1.00	0.81	800489	0.053
6.	Kaempferol	DAD	22.222	47.61	89.835	14.3	90.20	2.202	22.025	59	0.93	1.08	1058479	0.051

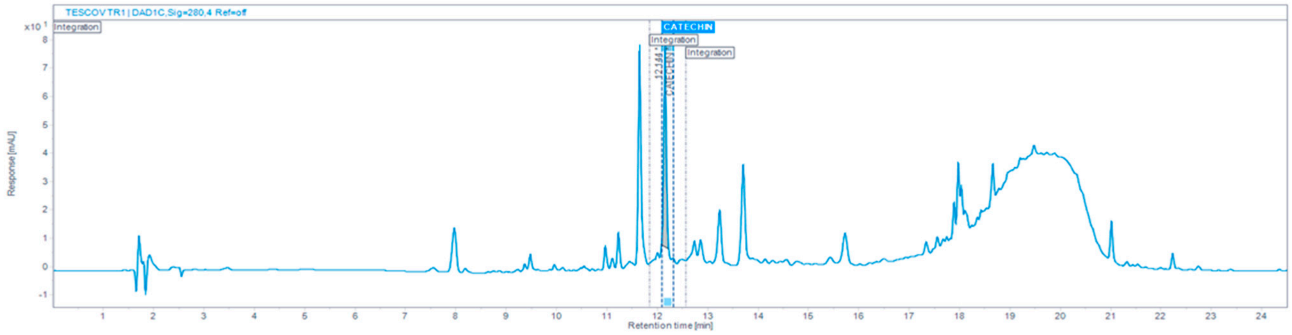

a

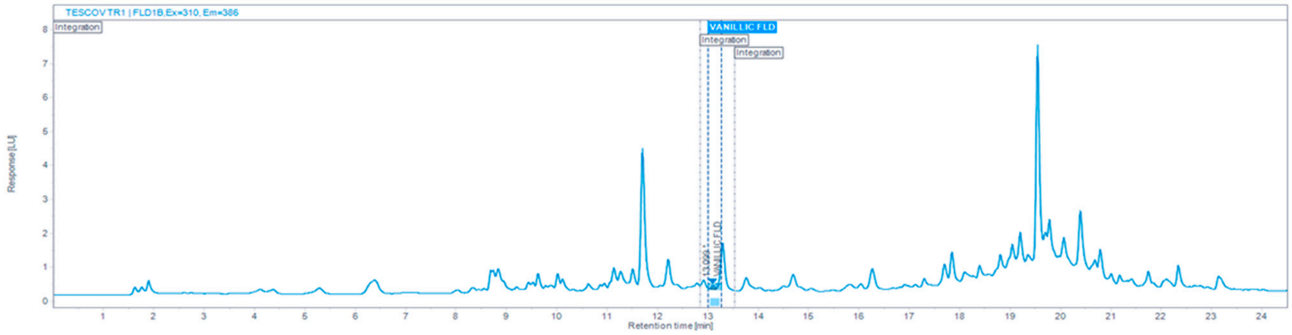

b

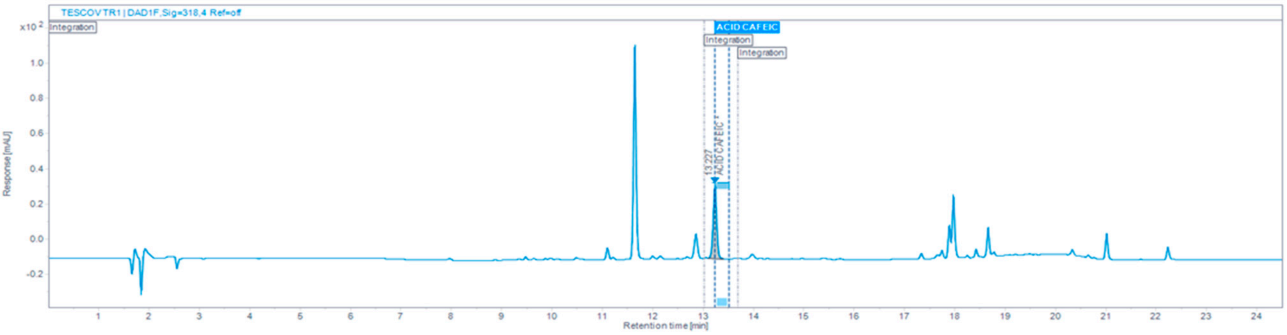

c

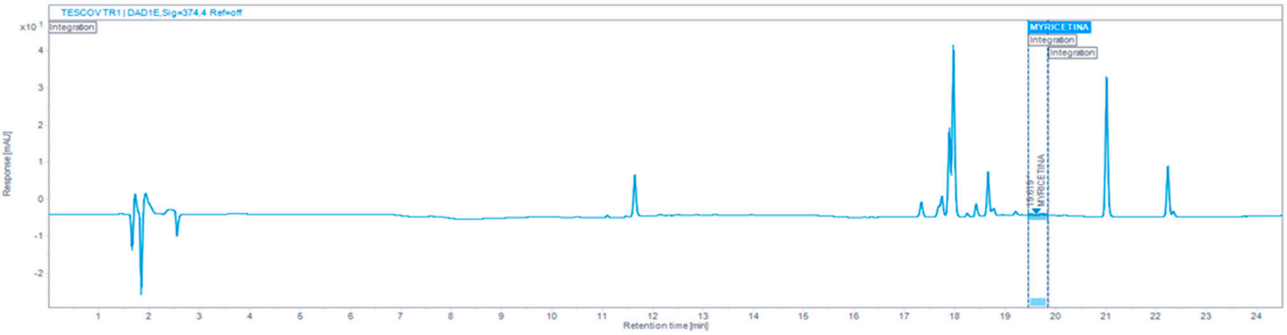

d

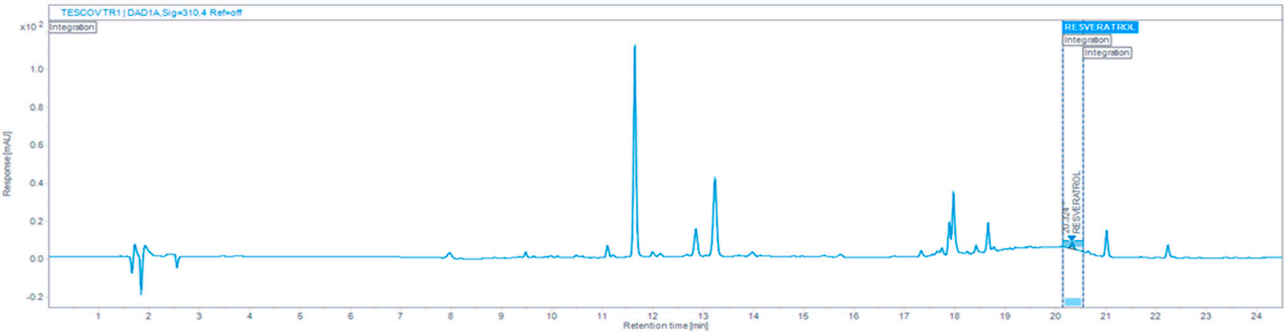

e

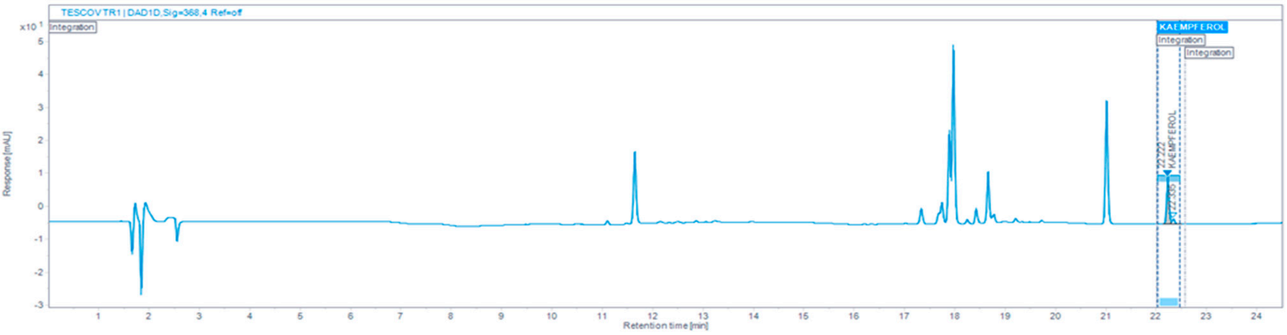

f

Figure S2. Chromatographic profile of the six polyphenols in the grape pomace extract of Tămâioasă Românească: (a) (+)catechin; (b) vanillic acid; (c) caffeic acid; (d) myricetin; (e) resveratrol; (f) kaempferol.

Table S9. Chromatographic data of the six polyphenols in the grape pomace extract of Fetească Neagră

No	Polyphenol	Detector	RT [min]	Area	Area 100%	Height	Height 100%	Amount [ppm]	Concentration [mg/kg]	S/N	Symmetry	Tailing	Plates EP	Width 50%[min]
1.	(+)Catechin	DAD	12.171	69.27	100.000	24.1	100.000	28.788	287.876	94	0.86	0.99	364369	0.047
2.	Vanillic acid	FLD	13.118	6.14	100.000	1.0	100.000	0.779	7.790	73	0.84	1.15	114955	0.091
3.	Caffeic acid	DAD	13.267	64.15	100.000	14.6	100.000	2.129	21.292	40	0.86	1.08	205464	0.069
4.	Myricetin	DAD	19.642	14.42	100.000	4.4	100.000	0.804	8.036	9	0.86	1.16	866622	0.050
5.	Resveratrol	DAD	20.341	44.13	100.000	12.4	100.000	1.193	11.935	33	0.86	0.87	825717	0.053
6.	Kaempferol	DAD	22.236	7.96	62.784	2.3	62.77	0.354	3.545	5	0.95	1.01	1017953	0.052

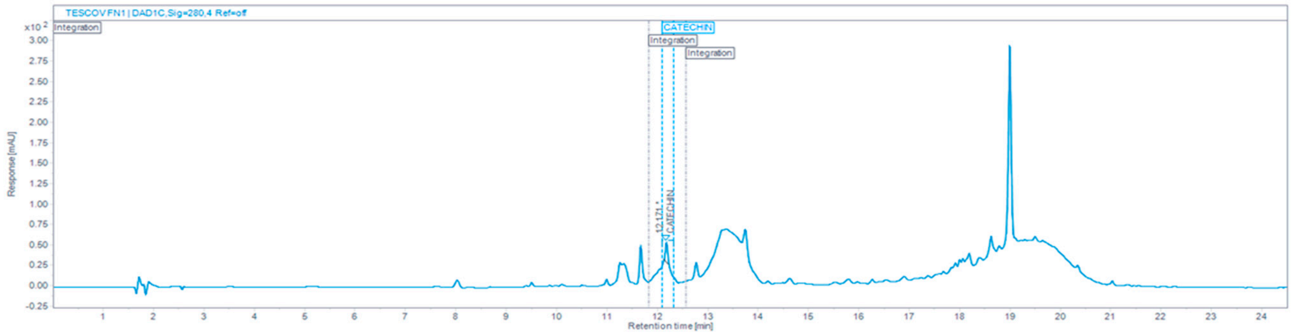

a

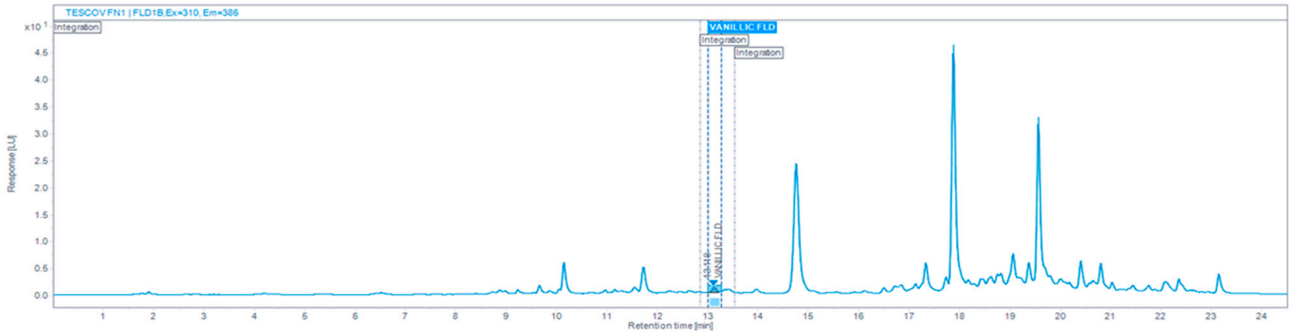

b

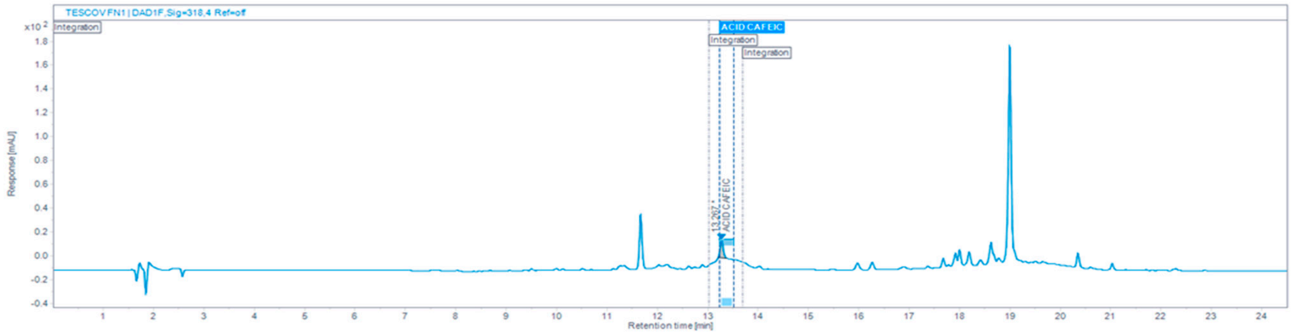

c

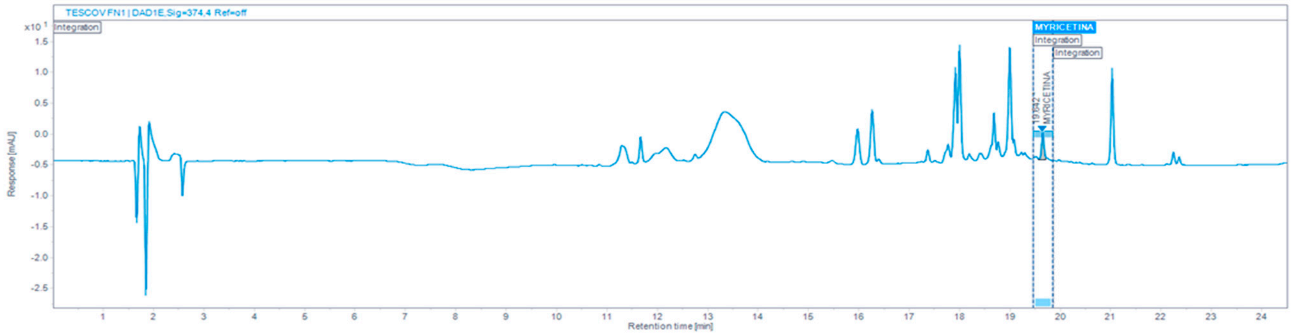

d

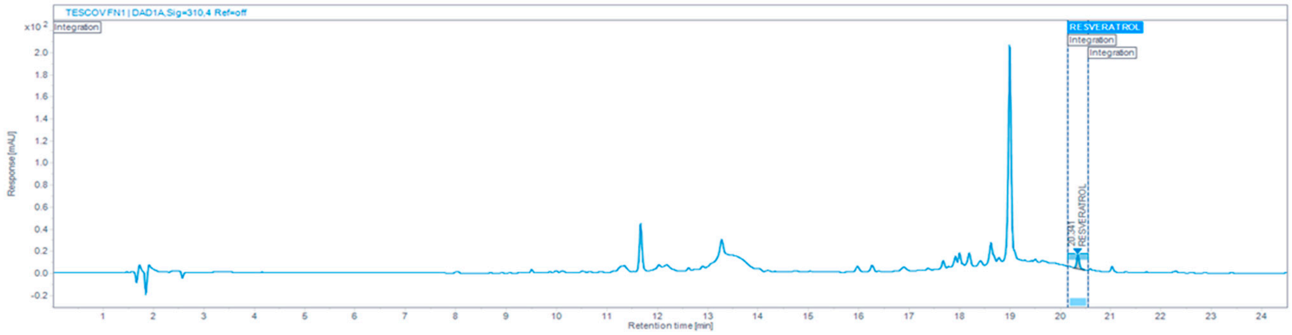

e

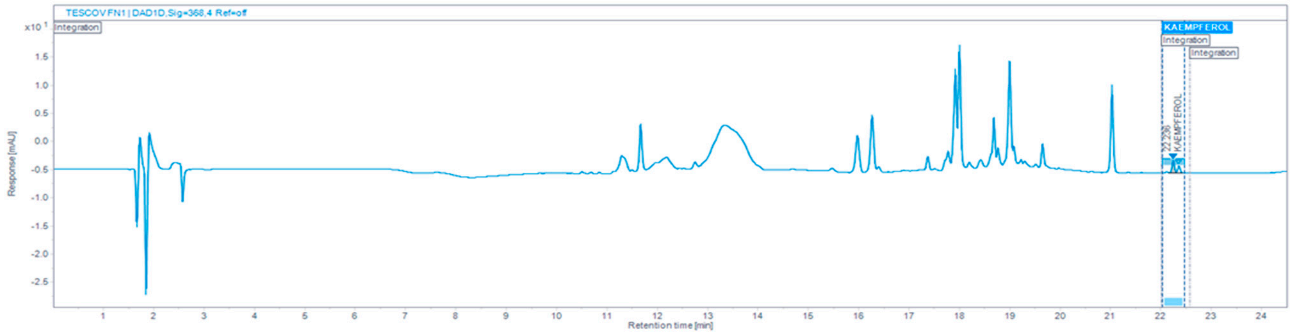

f

Figure S3. Chromatographic profile of the six polyphenols in the grape pomace extract of Fetească Neagră: (a) (+)catechin; (b) vanillic acid; (c) caffeic acid; (d) myricetin; (e) resveratrol; (f) kaempferol.

Table S10. Chromatographic data of the six polyphenols in the grape pomace extract of Negru de Drăgășani

No	Polyphenol	Detector	RT [min]	Area	Area 100%	Height	Height 100%	Amount [ppm]	Concentration [mg/kg]	S/N	Symmetry	Tailing	Plates EP	Width 50%[min]
1.	(+)Catechin	DAD	12.157	329.23	100.000	110.8	100.00	136.762	1367.623	1116	0.92	0.97	342489	0.049
2.	Vanillic acid	FLD	13.097	7.07	100.000	1.2	100.00	0.894	8.945	120	0.79	1.16	119868	0.089
3.	Caffeic acid	DAD	13.246	124.20	100.000	25.8	100.00	4.135	41.353	107	0.94	1.27	182091	0.073
4.	Myricetin	DAD	19.632	19.79	87.753	6.1	89.04	1.104	11.042	17	0.88	1.12	888220	0.049
5.	Resveratrol	DAD	20.331	63.15	100.000	17.8	100.00	1.717	17.168	72	0.84	0.87	810529	0.053
6.	Kaempferol	DAD	22.228	19.83	81.814	5.9	82.25	0.907	9.075	16	0.95	1.09	1046818	0.051

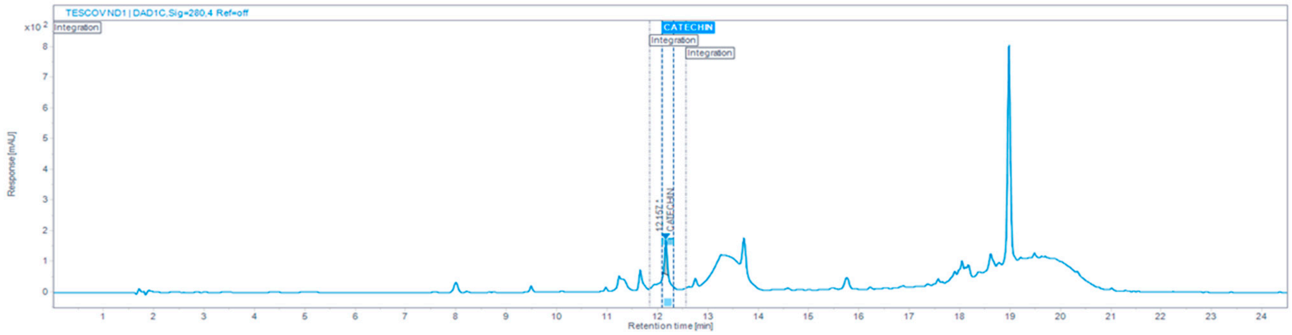

TESCOVND1 | DAD1C,Sig=280,4 Ref=off
CATECHIN
Integration
Response [mAU]
Retention time [min]

a

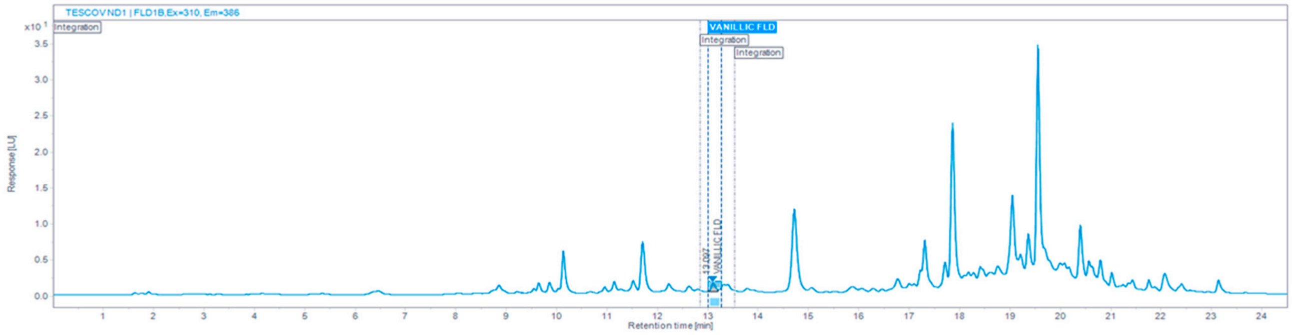

TESCOVND1 | FLD1B,Ex=310, Em=386
VANILLIC FLD
Integration
Response [LU]
Retention time [min]

b

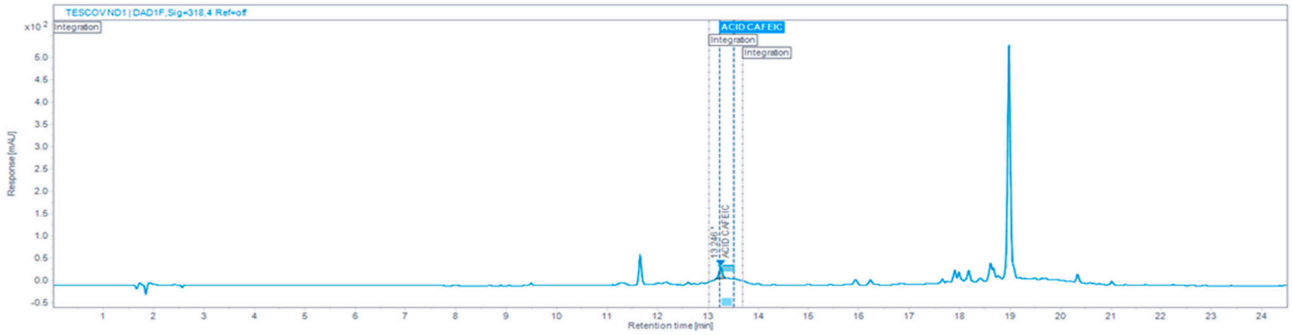

TESCOVND1 | DAD1F,Sig=318,4 Ref=off
ACID CAFEIC
Integration
Response [mAU]
Retention time [min]

c

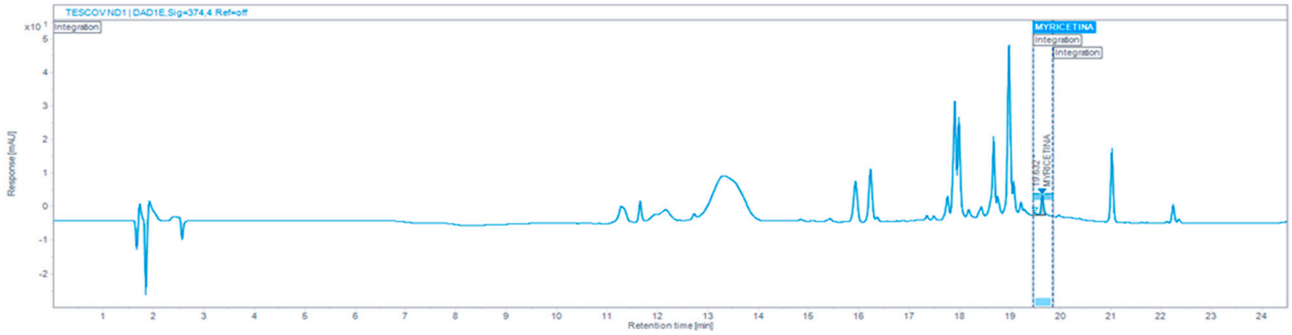

TESCOVND1 | DAD1E,Sig=374,4 Ref=off
MYRICETINA
Integration
Response [mAU]
Retention time [min]

d

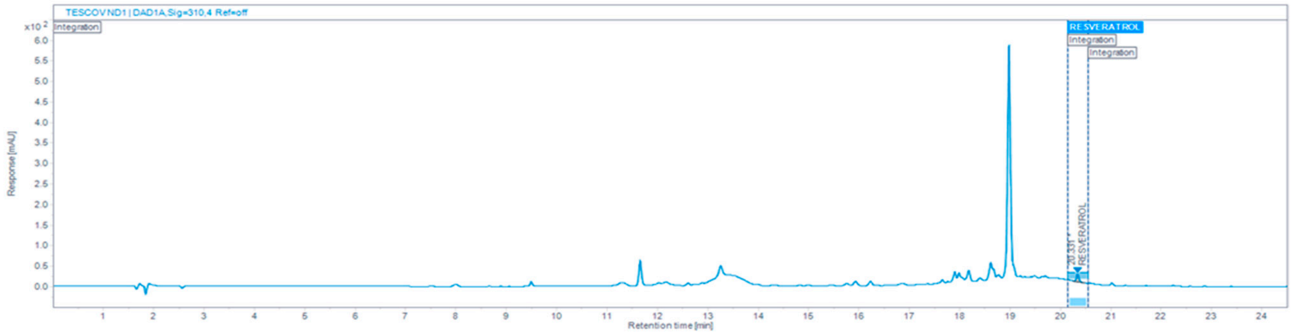

TESCOVND1 | DAD1A,Sig=310,4 Ref=off
RESVERATROL
Integration
Response [mAU]
Retention time [min]

e

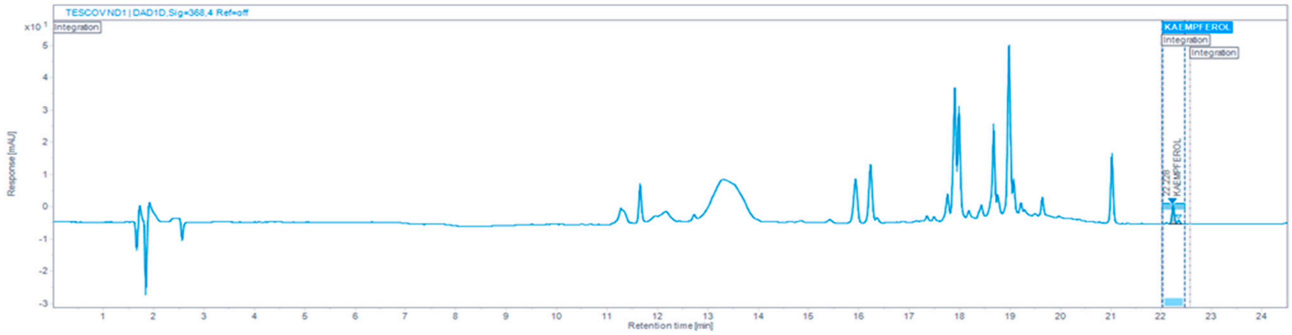

f

Figure S4. Chromatographic profile of the six polyphenols in the grape pomace extract of Negru de Drăgășani: (a) (+)catechin; (b) vanillic acid; (c) caffeic acid; (d) myricetin; (e) resveratrol; (f) kaempferol.
